# Rapid automated 3-D pose estimation of larval zebrafish using a physical model-trained neural network

**DOI:** 10.1371/journal.pcbi.1011566

**Published:** 2023-10-23

**Authors:** Aniket Ravan, Ruopei Feng, Martin Gruebele, Yann R. Chemla

**Affiliations:** 1 Center for Biophysics and Quantitative Biology, University of Illinois at Urbana-Champaign, Urbana, Illinois, United States of America; 2 Center for the Physics of Living Cells, University of Illinois at Urbana-Champaign, Urbana, Illinois, United States of America; 3 Department of Chemistry, University of Illinois at Urbana-Champaign, Urbana, Illinois, United States of America; 4 Department of Physics, University of Illinois at Urbana-Champaign, Urbana, Illinois, United States of America; University of California Santa Barbara, UNITED STATES

## Abstract

Quantitative ethology requires an accurate estimation of an organism’s postural dynamics in three dimensions plus time. Technological progress over the last decade has made animal pose estimation in challenging scenarios possible with unprecedented detail. Here, we present (i) a fast automated method to record and track the pose of individual larval zebrafish in a 3-D environment, applicable when accurate human labeling is not possible; (ii) a rich annotated dataset of 3-D larval poses for ethologists and the general zebrafish and machine learning community; and (iii) a technique to generate realistic, annotated larval images in different behavioral contexts. Using a three-camera system calibrated with refraction correction, we record diverse larval swims under free swimming conditions and in response to acoustic and optical stimuli. We then employ a convolutional neural network to estimate 3-D larval poses from video images. The network is trained against a set of synthetic larval images rendered using a 3-D physical model of larvae. This 3-D model samples from a distribution of realistic larval poses that we estimate a priori using a template-based pose estimation of a small number of swim bouts. Our network model, trained without any human annotation, performs larval pose estimation three orders of magnitude faster and with accuracy comparable to the template-based approach, capturing detailed kinematics of 3-D larval swims. It also applies accurately to other datasets collected under different imaging conditions and containing behavioral contexts not included in our training.

## Author summary

Larval zebrafish swimming has been studied extensively in 2-D environments, which are restrictive compared to natural 3-D habitats. To enable rapid yet robust capture of 3-D poses, we collect three orthogonal video projections of swim behaviors in several behavioral settings and fit poses to a physical model. We then use the physical model to generate an auto-annotated stream of synthetic poses to train a convolutional neural network. The network model performs highly accurate pose predictions on over 600 real swim bouts much faster than a physical model fit. Our results show that larvae frequently exhibit motions inaccessible in a 2-D setup. The annotated dataset could be used by ethologists studying larval swimming dynamics, and by the machine learning community interested in multi-dimensional time series and 3-D reconstruction. Using the ability to render images with multiple synthetic poses, our method can be extended to collective behavior.

## Introduction

Neuroethologists have long sought an understanding of the neural basis of behavior, through multiple model organisms offering varying degrees of complexity of their nervous system. An important step in achieving this goal is to develop quantitative tools to identify distinct behaviors objectively [1–8]. This step requires accurate tracking of an organism’s pose as a function of time. Historically, this has been done by manually labeling the points of interest on the body, which is a laborious process and subject to errors. However, over the last decade, it has become increasingly possible to automate several image analyses tasks, including animal pose estimation with human-level accuracy [9–19]. These advances can be credited to the success of artificial neural networks in conjunction with development of graphical processing units (GPUs). Artificial neural network models can be tuned for pose estimation, given examples of image data with a priori annotated poses. Since these annotations are created by manual human labelling in most cases, the network’s accuracy still is bound by that of the source generating the annotations.

*Danio rerio* (zebrafish) larvae are a popular choice of model vertebrate organism and their swimming behavior in response to several environmental stimuli has been studied extensively over the last several decades [5,6,20–29]. These studies are mostly restricted to 2-D, where the larva is constrained to swim in a shallow (2–3 mm height) medium and imaged using a single overhead camera. Thus, these measurements lack a complete three-dimensional picture of larval swimming dynamics. Moreover, the depth available for swimming is not only smaller than the length of the organism (3–4 mm long at 6–7 days post fertilization (dpf)), but also severely restrictive compared to its native environment [30]. It is plausible that this constraint influences the swimming motion of the larva. Recent literature emphasizing the need to study naturalistic animal behaviors [31,32] makes a strong case for tools to track the 3-D pose of larval zebrafish.

**Fig 1:**
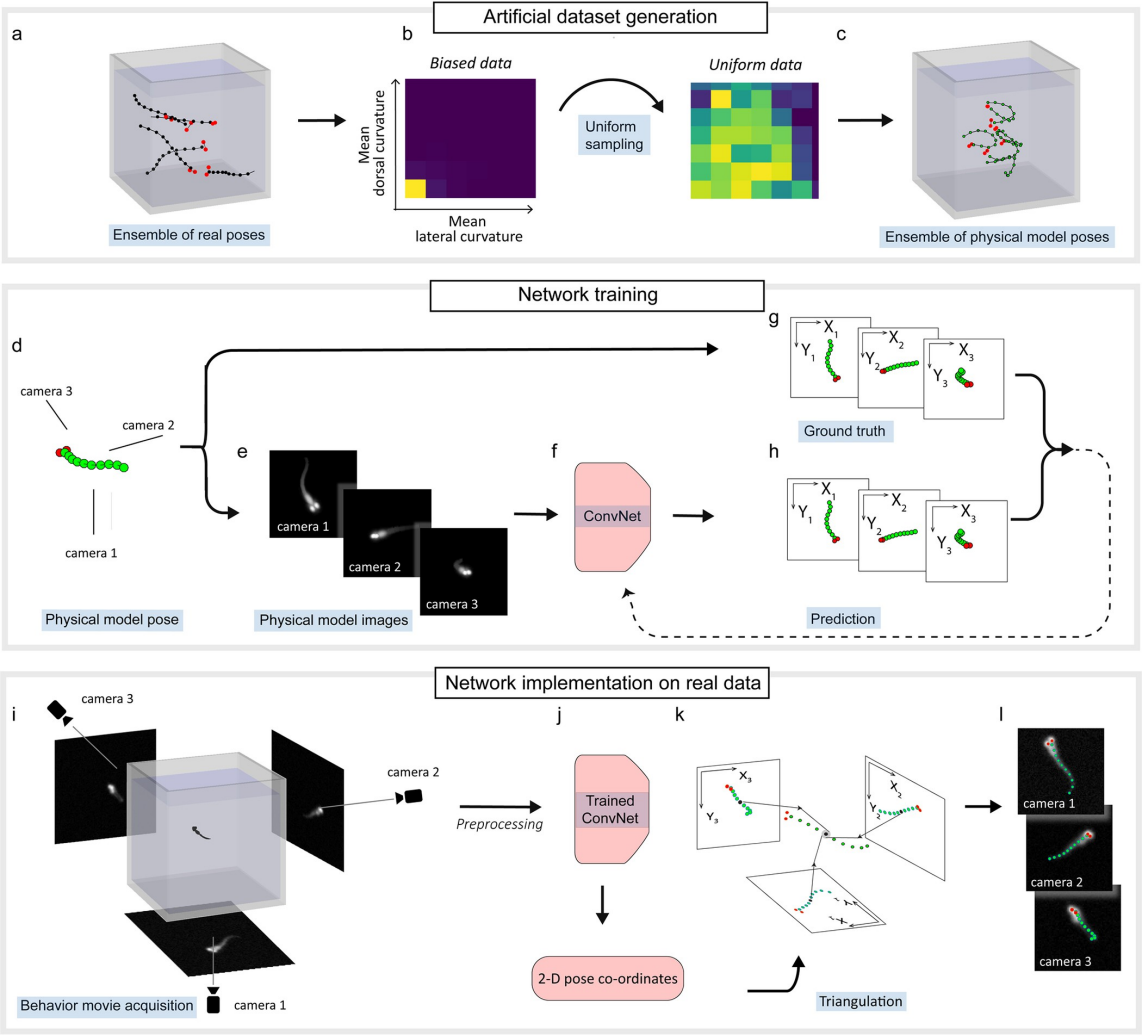
Convolutional neural network model trained on physical model images performs fast and accurate pose estimation on real images. (a) *Ensemble of real poses*: Larval poses in real images were estimated using a template-based pattern recognition approach, and a subset of poses estimated with high accuracy were selected for generating an ensemble of real poses (*N* = 35714). (b) *Biased data*: The ensemble of real poses is biased towards larval shapes with nearly straight backbones, at (0,0) when visualized on a 2-D space of mean lateral and dorsal body curvature. *Uniform data*: A subset of 2500 poses is sampled from the ensemble in b, such that larval shapes with different lateral and dorsal curvature are represented uniformly. (c) *Ensemble of physical model poses*: *N* = 500,000 poses are generated from the uniform data by sampling its backbone angles and other parameters, and from the training dataset for the neural network model. (d) *Physical model poses* are used in (e) to render physical model 2-D ‘camera view’ images for input to the network model. (f) The *convolutional neural network* model receives images from (e) in its three input channels. (g) ‘*Ground truth’* annotation: 2-D pose annotations are generated by mapping the 3-D pose using a nonlinear projection function estimated during camera calibration. (h) The *predictions* of the neural network are 3×12 2-D pose coordinates. The network parameters are optimized by using the loss function defined as the root mean squared error between the network *predictions* and the *ground truth* annotations. (i) *Behavioral movies* recorded by three orthogonal cameras are preprocessed by cropping and background subtraction. (j) The *trained convolutional network* turns preprocessed images into 2-D pose coordinates of the recorded larva. (k) *Triangulation*: 3-D pose coordinates are triangulated using the 2-D pose coordinates and the empirically determined projection function. (l) The 2-D projections of the estimated pose are superimposed on a set of real images for visual inspection.

These limitations motivated us to develop a fast and accurate technique for estimation of the poses (position, orientation, and shape of the backbone) of larval zebrafish swimming in a 3-D environment. We collected videos of individual larvae swimming in a cubic glass tank of spatial dimensions an order of magnitude larger than the length scale of a larva. Videos were captured by three orthogonal cameras, calibrated to account for non-linearities due to refraction (**Materials and Methods: (a) Instrumentation:** *Camera calibration*). Our fully automated method of pose estimation relies on a convolutional network trained on a set of digitally rendered larval images with auto-annotated pose (**Fig 1**). We refer to these rendered images as “physical model images” henceforth.

The physical model images are rendered using a voxel-based physical model of the larva, where voxels are mapped into pixels of three orthogonal camera views using 3-D-to-2-D projection parameters obtained during camera calibration. The poses used to generate these images are sampled from a distribution of poses estimated a priori using a computationally expensive template-based pose estimation (**Materials and Methods: (b) Template-based pose estimation** and **S1 Fig**). The use of annotated physical model images obviates the need for human labelling, which in this context is not merely an inconvenience, but also extremely difficult. The challenges in human labelling are due to occlusions and the necessity to label consistent points on the organism across the three views and across different frames on the larval backbone. Such consistency in labelling is made challenging by the lack of any natural fiducial markers on the organism. A similar approach involving digitally generated annotated images via the use of real images and affine transforms was recently developed [12] for pose estimation of *C*. *elegans*. However, this approach cannot be extended to the case of a 3-D larva where larval images are frequently characterized by self-occlusions. Moreover, zebrafish larvae consist of more complex features than worms, like eyes, head and belly, whose motion cannot be accounted for by using affine transforms of their 2-D projections.

Here, we record larval images in three commonly studied behavioral contexts: free swimming, acoustic startle, and dark flash (**S3 Fig**) and use our physical model-trained neural network tool to estimate larval poses in these recordings. We then evaluate the convolutional neural network’s performance by comparing the correlation coefficient between raw images and the physical model images resulting from the model’s predictions. The neural network model produces results much faster than the template-based pattern recognition technique and with equal or better accuracy. Our measurements reveal a rich set of 3-D kinematics of larval swims not accessible in 2-D experiments.

Our work can be summarized by three modular tools presented here as a single technique for larval pose estimation:

1. a neural network model for fast and accurate 3-D pose estimation of zebrafish larvae;
2. a large collection of diverse larval 3-D poses and the corresponding raw images, of interest to the ethologists, zebrafish researchers, and machine learning community;
3. a technique to render realistic larval images which can be used to generate an arbitrarily large training dataset to train a neural network to model other datasets with behavioral contexts different from any of our training datasets.

A visualization of a 3-D reconstructed larval swim bout from an acoustic startle experiment is presented in **S1 Video**.

## Results

### Convolutional neural network model trained on physical model images performs fast and accurate pose estimation on real images

#### Experimental design

Fish swimming measurements were carried out on 6–9 days-old larvae a few millimeters in length in a cubic glass tank of dimensions 7 × 7 × 7 cm (see **S3A Fig** and **Materials and Methods: (a) Instrumentation**). Movies of the zebrafish were obtained using 3 synchronized high-speed cameras taking orthogonal images of the fish tank at 500 frames per second (fps) (**S1A Fig**). While two orthogonal axes are in principle sufficient to reconstruct the position of simple rod-like shapes, three axes provide redundancy to correct for small errors due to water refraction, and additional information for more complex shapes, such as a highly bent backbone that obscures parts of itself from certain view angles. We calibrated the cameras taking into account refraction at the water-glass and glass-air interfaces using a dot grid. We also estimated a projection function, modelled as a cubic polynomial, that maps 3-D coordinates in the tank to 2-D pixel coordinates for each camera (see **Materials and Methods: (a) Instrumentation:** *Camera calibration*: *Mapping from lab to camera coordinates* and **S4 Fig**). A 3 × 3 × 3 cm cubic region in the center of the tank overlapped the field of view of all the three cameras. For all our experiments, we exclusively recorded larvae swimming in this central cubic region of the tank to avoid edge effects from interactions between the fish and the walls. We used three orthogonally placed and diffused high-power (50 W) near-infrared LEDs (850 nm) for illumination outside the larvae’s wavelength sensitivity [33]. This illumination did not heat the water significantly yet compensated for the high frame rate necessary to capture maneuvers reliably and the small optical aperture (large *f*-ratio) necessary in order to achieve a large depth of field. For dark flash experiments, a white LED was used in addition to the infrared illumination, to provide the dark flash stimulus (see **Materials and Methods: (a) Instrumentation**: *Experimental Setup* and **S3B Fig**).

Zebrafish larvae were placed in the tank and allowed to swim in free swimming, acoustic startle, and dark flash contexts. We collected a total of 630 movies of larvae swimming in 3-D, with at least 100 movies from each type of experiment (free swimming– 303, acoustic startle– 162, dark flash– 165). Free swimming videos were recorded for a duration of 20 seconds each at an interval of 3 minutes. For acoustic startle experiments, an acoustic stimulus was generated by dropping a weight from a fixed height onto the platform mounting the glass tank (**S3C Fig**). A dark flash stimulus was generated by turning off a white LED placed near the tank for 10 s (**S3B Fig**). To increase measurement throughput, both types of stimuli were triggered automatically whenever the three cameras detected at least one larva in the 3 × 3 × 3 cm imaging region. Consecutive stimuli were separated by at least 4 minutes in time to prevent habituation of the larvae to the stimulus. This inter-stimulus interval (ISI) is significantly larger than an ISI of 15 seconds reported in Ref. [34] to induce habituation to acoustic startle stimuli. We found in our experiments that an ISI of 4 minutes was sufficient to obtain consistent larval responses to dark flashes in the form of O-bends [34]. On average, a video for acoustic startle and dark flash experiments was recorded every 10 minutes for an interval of 4 seconds. The stimulus was provided 1 second after beginning the recording, providing sufficient time (3 seconds) to capture the response. The setup is described in detail in **Materials and Methods: (a) Instrumentation** and **S3 Fig**.

#### The 3-D physical model of a larval zebrafish

We represented a larva using a physical model. The physical model requires 12 3-D coordinates to define a larval pose: 10 equally spaced coordinates along its backbone, and 2 coordinates defining the centroid of its eyes. The physical model is composed of 9 segments connected by flexible hinges, and includes a larval anterior composed of head, eyes, and ventral region located by the first two segments and a posterior composed of seven tail segments (**S1C Fig**). The coordinates of the physical model are then used to render realistic 2-D larval projections (see **Materials and Methods: (b) Template-based pose estimation**: *Rendering larval projections*, *Physical model of the larva* for more details). We refer to these projections as “physical model images”. We previously found that 9 backbone segments were sufficient to converge behavioral analysis in 2-D [23].

A definition of the physical model in terms of the angles between its consecutive segments is more suitable for direct quantitative analysis of behavior. Thus, we also formulated an equivalent description of the physical model in 3-D by specifying a set of adjustable parameters encoded in the 22-parameter vector **p** = (*x*_0_, *y*_0_, *z*_0_, *θ*_0_, Δ*θ*_1_,… Δ*θ*_8_, *φ*_0_, Δ*φ*_1_,… Δ*φ*_8_, *γ*_0_) and a fixed parameter, *l* (fish length). *x*_0_, *y*_0_, *z*_0_ determine the centroid of the head relative to the lab reference frame *x*_lab_, *y*_lab_, *z*_lab_, and the Euler angles *θ*_0_, *φ*_0_, *γ*_0_ determine the head orientation (yaw, inclination, and roll, respectively) in the lab reference frame. **ϴ** **=** (Δ*θ*_1_,… Δ*θ*_8_, Δ*φ*_1_,… Δ*φ*_8_) is the set of angles that give the orientation of each body segment and determines the fish shape. Here, Δ*θ*_*i*_ and Δ*φ*_*i*_ are the bending angles for the (*i*+1)^th^ segment in the lateral and dorsal planes, respectively, as measured in the fish reference frame *X*, *Y*, *Z*, defined by the head orientation *θ*_0_, *φ*_0_, *γ*_0_ (see **S1C Fig** for angle nomenclature).

A larval pose can thus be uniquely identified using either of the following equivalent representations:

1. 3-D pose coordinates: 12 3-D coordinates in the cartesian lab reference frame *x*_lab_, *y*_lab_, *z*_lab_ that define the physical model;
2. 2-D projection pose coordinates: 2-D projections of the 3-D pose coordinates obtained using the projection function;
3. 22-parameter vector **p** and *l*.

### Generation of the training and validation datasets

We sought to train a convolutional neural network model to estimate larval poses from pre-processed larval images (see **Materials and Methods: (c) Neural Network pose estimation**: *Preprocessing training dataset*) captured by the three cameras. Our approach is summarized in **Fig 1** (also see **S2 Fig**), and consists of three steps: generation of a synthetic training dataset, training of the convolutional neural network, and implementation of the network on real larval images. In the last step, the network maps the larval images to three 2-D projection pose coordinates, which can then be triangulated to obtain the 3-D pose coordinates using the projection function in a single post-processing step.

**Fig 1A–1C** illustrates the procedure for generating training and validation datasets. These datasets consist of physical model images used as the neural network inputs and their corresponding 2-D projection pose coordinates as the ground truth annotations. We rendered 500,000 physical model images and computed their corresponding 2-D projection pose coordinates using an ensemble of physical model poses (see *Ensemble of physical model poses* in **Fig 1C**), which is a large set of realistic larval poses generated using a probabilistic model informed from a much smaller ensemble of real larval poses (see **Fig 1A and 1B**). We split the training and validation dataset in a 9:1 ratio while training the convolutional neural network model. The lack of human intervention in generating this training and validation dataset results in efficient generation of accurate labels.

The ensemble of real larval poses (**Fig 1A**) required to generate the training dataset comprises 35714 distinct real larval poses. It is obtained by first using a computationally expensive template-based pose estimation performed in over 424 larval video recordings (41756 frames), which is a fraction our total dataset of 630 videos (63181 frames) (see **S1 Fig** and **Materials and Methods: (b) Template-based pose estimation**). We then select a subset of the poses with a pose estimation score of at least 0.9 (see **Results: *Model evaluation*** for a definition of pose estimation score). Briefly, we perform a search in the 22-parameter space of the vector **p**. For any given **p** and *l*, the physical model is uniquely determined, and physical model images are rendered using the projection function (see **Materials and Methods: (b) Template-based pose estimation:** *Physical model of the larva*). We optimize **p** (see **Materials and Methods: (b) Template-based pose estimation:** *Optimization*) by minimizing the sum of squared difference between the physical model images and the pre-processed data captured by the orthogonal cameras (**S1B–S1H Fig)**.

Finally, the ensemble of real larval poses is used to generate a realistic and unbiased ensemble of physical model poses using a probabilistic model (**Fig 1A–1C**). This approach is adopted mainly for two reasons: (1) We observe that the majority of real larval poses from swim recordings consist of a nearly straight backbone poses, and this biased ensemble is thus not ideal as a training dataset. The distribution of *mean lateral curvature* <|Δ*θ*_i_|> and *mean dorsal curvature* <|Δ*φ*_i_|>, where *iϵ*{1,2,…,7,8} (**Fig 1B** *Biased data*) illustrates this point, showing that most swim bouts are distributed near <|Δ*θ*_i_|> = 0 and <|Δ*φ*_i_|> = 0). (2) A probabilistic model informed from the ensemble of real poses allows us to generate an arbitrarily large number of distinct physical model poses. This model is obtained in two steps. First, we use a kernel density estimate to create a uniform data set of 2500 larval poses, sampled from the entire set of 35714 poses in the ensemble of real larval poses, such that larval shapes of different lateral curvature <|Δ*θ*_i_|> and dorsal curvature <|Δ*φ*_i_|> are uniformly represented (depicted in **Fig 1B** as the transformation from *Biased data* to *Uniform data* and described in **Materials and Methods: (c) Neural network pose estimation:** *Generation of ensemble of physical model poses*). The size of the uniform data (*N* = 2500) was chosen as a trade-off between the diversity of larval poses and computational complexity. Next, we again use a kernel density estimate to approximate the probability distribution ℘_*angle*_ (**ϴ**, *φ*_0_, γ_0_) of all the 2500 poses in the uniform data. Since the distribution ℘_*angle*_ is built in the (**ϴ**, *φ*_0_, *γ*_0_) space, any correlations existing between Δθ_*i*_, Δ*φ*_i_ *φ*_0_ and *γ*_0_ in the 2500 real poses are represented by the distribution. A physical model pose can be generated using a probabilistic model, which samples a vector (**ϴ**, *φ*_0_, γ_0_) from ℘_*angle*_ and assigns to it (*x*_0_, *y*_0_, *z*_0_) within the glass tank’s imaging volume and *θ*_0_ in the range (−*π*, *π*] by sampling from a uniform distribution. This probabilistic model is used to sample 500,000 novel and distinct larval poses, forming the ensemble of physical model poses (**Fig 1C**). **S2 Video** shows a random selection of 100 physical model poses.

### Training an artificial neural network model

The procedure for training the convolutional neural network is illustrated in **Fig 1D–1H**. First, for each physical model pose in the ensemble (**Fig 1D**), physical model images are rendered using the voxel-based model (**Fig 1E** and **Materials and Methods: (b) Template-based pose estimation**: *Physical model of the larva*, *Rendering larval projections*). These images have a modest resolution of 141x141, to accommodate larval projections of all sizes seen in our data. The larval projections are then displaced from the center of the image at random by 0–20 pixels and Gaussian white noise is added to mimic the experimental data (see **Materials and Methods: (c) Neural network pose estimation:** *Preprocessing training dataset* for more details). At the same time, the 2-D projection pose coordinates are computed using the projection function and the 3-D pose coordinates, forming the ground truth annotations for the three images (**Fig 1G**).

We use a convolutional neural network (**Fig 1F**) inspired by the recent success of Residual Networks [35] on various pattern recognition tasks, including animal pose estimation [12,16]. We leverage the highly effective feature-extraction power of residual blocks for our application. Our network consists of two modules: an encoder and a decoder. The encoder consists of four bottleneck residual blocks as implemented in Ref. [35] with 32, 64, 128, and 256 channels respectively. The decoder consists of three fully connected layers of dimensions 1×288, 1×144 and 1×72. The convolutional neural network model is described in greater detail in **Materials and Methods: (c) Neural network pose estimation:** *Convolutional neural network model*.

The neural network accepts the three 2-D physical model projections corresponding to the three camera views as three input channels (**Fig 1E and 1F**). The network’s prediction is designed to be 3x12 2-D coordinates (10 backbone coordinates plus 2 eye coordinates for each projection), forming the predicted 2-D projection pose coordinates (**Fig 1H**) for each camera view. The network’s parameters are tuned by minimizing a loss function defined as the sum of squared errors between the predicted 2-D projection pose coordinates and the ground truth annotations, summed over all the three camera views. The loss function is made symmetric with respect to the two eyes (see **Materials and Methods: (c)** *Neural network pose estimation* for more details on the loss function). Since the network model processes the three channels simultaneously, information between the three larval views is coupled for an accurate prediction of the 2-D projection pose coordinates. Thus, the lack of knowledge about the location of the larval backbone due to self-occlusion in one view can be compensated by other view(s) in which there is no self-occlusion to generate an accurate predicted annotation.

### Pose inference using the artificial neural network model

The procedure for performing pose estimation using the trained convolutional neural network is illustrated in **Fig 1I–1L**. The larval pose estimation is performed independently for each frame. The raw data is first pre-processed by performing background subtraction and cropping a window of 141×141 pixels around the three images of a larva, followed by median filtering (see **S7 Fig** and **Materials and Methods: (b) Template-based pose estimation**: *Preprocessing*; resulting images shown in **Fig 1I**). These pre-processed images are passed to the network (**Fig 1J**) trained on physical model images to generate the 2-D projection pose coordinates (**Fig 1L**). The 12 points of the larval pose are triangulated in parallel using the inferred 2-D projection pose coordinates and the projection function (**Fig 1K**). The triangulation is trivial for the backbone coordinates. The eye coordinates are triangulated by taking into account the symmetry of the loss function with respect to the eye indices (see **Materials and Methods: (c) Neural network pose estimation**: *Triangulation of 3-D pose coordinates*). We further improve the pose prediction by fitting a spline through the estimated 3-D backbone coordinates and computing 10 equally spaced coordinates along the arc length [36]. This step ensures that the points estimated along the backbone are equidistant and the angles between the segments of the physical model (Δ*θ*_i_, Δ*φ*_i_) are consistently defined.

### Model evaluation

**Fig 2:**
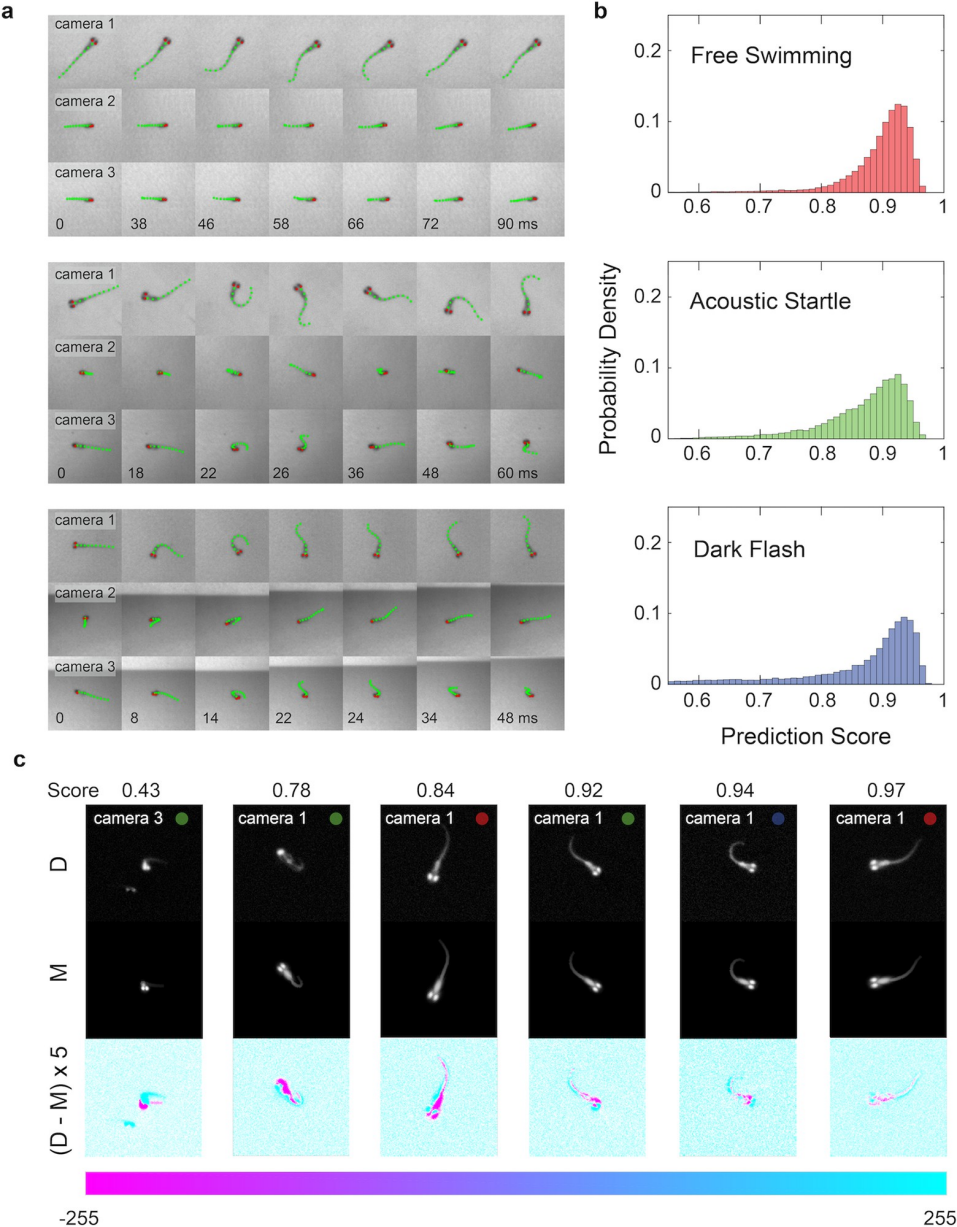
Evaluation of the predicted poses on real data. (a) Example sequences of raw images from the three camera views and superimposed 2-D projected poses from the neural network: ten 2-D backbone coordinates (green) and two 2-D centroids of eyes (red). Top block: free swimming; middle block: acoustic startle; bottom block: dark flash. Larval lengths range between 3.5 and 4 mm. (b) Histograms of the pose prediction scores (68181 frames in 630 movies overall) for the three behavioral experiments illustrated in (a): free swim (red), acoustic startle (green), dark flash (blue). ‘1’ corresponds to a perfect pose prediction. (c) Comparison between experimental and model images representative of the prediction scores in (a). Colored dots (red, green, blue) denote the experimental context for each image. Only one of the three views of the fish is shown. D: experimental Data collected with the three-camera system, M: Model images rendered using the voxel-based model. (D-M)×5: Difference image of the experimental Data and Model, amplified by a factor of 5. Pixel values in the range [–255, 255] of the amplified difference image can be interpreted by the colorbar below.

**Fig 2A** shows the results of the larval pose prediction for representative bouts in free swimming, acoustic startle, and dark flash experiments. Selected movie frames from the 3 cameras are displayed, with the coordinates of the physical model superimposed (10 backbone coordinates in green circles, 2 eye coordinates in red circles). The neural network model performs larval pose prediction approximately three orders of magnitude faster than the template-based pose estimation. Pose prediction using the neural network model takes ~300 milliseconds per frame, significantly faster than the 10–12 minutes per frame for template-based pose estimation (**Materials and Methods: (b) Template-based pose estimation**). Thus, pose prediction using a neural network model is a much more practical approach compared to the template-based approach. We next asked how the pose prediction accuracy of the neural network’s model compares to that of the template-based approach, using a quantitative assessment of the accuracy of the two approaches.

To evaluate our neural network model, we used its output pose to render physical model images and compared them to the corresponding three physical model input images. We computed the Pearson’s correlation coefficient between each of the three pairs of input and output images. The network’s pose prediction score for a given frame is defined as the minimum of the three correlation coefficients. (We observed that the predictions scores were on average higher for camera 3, which we attribute to better calibration.) Before computing the correlation coefficients, a binary mask was obtained by segmenting the physical model images. The correlation coefficient was only calculated for non-zero pixel locations occupied by this mask. Since the input images have a noisy background and the output images have a black background, there is no correlation between the background pixels of the pair of images. Using a mask ensures that the value of the correlation coefficient is determined mainly by the pixels comprising the images of the larval body, and not those that make up the background. **Fig 2B** shows the network’s pose prediction score on our entire free swimming, acoustic startle, and dark flash dataset comprising 630 movies (68181 frames). The quality of the pose estimate can be interpreted by comparing the physical model image and the recorded image for different values of the correlation coefficient (see **Fig 2C**). Thus, the prediction score distribution can be used to assess the relative “goodness of fit” for pose predictions within a dataset. On average we observe that highly bent poses generate slightly lower prediction scores than straighter poses, which could reflect limitations of the 9-segment physical model in describing highly bent poses.

We trained the network with further datasets to evaluate its robustness to variability of the input data. We generated independent training datasets, using 100% (*N* = 35714) vs. 25% (*N* = 8928) of the poses in the ensemble of real poses, and using poses from each experimental context only (free swimming poses, *N* = 9536; acoustic startle poses, *N* = 13320; and dark flash poses, *N* = 12858) in the ensemble of real poses. We also evaluated the accuracy of the template-based pose estimations as a comparison. Evaluations were performed over all 424 swim bouts (41756 frames) for which the computationally expensive template-based pose estimation was performed. We find that the trained convolutional neural network model (mean pose prediction score = 0.91) on average performs as well or better than the template-based pose estimation technique (p < 0.01; mean pose prediction score = 0.90) (**S8 Fig** and **Materials and Methods: (e) Statistical analysis**). Thus, the neural network model is superior to the template-based pose estimation in speed without sacrificing accuracy. We also find that reducing the amount of experimental data (25% of all poses) used in the training dataset does not deteriorate the performance of the network model (pose prediction score = 0.91). Training datasets generated from only acoustic startle or dark flash poses perform similarly well (pose prediction score = 0.91; summarized in **S2 Table**). Using only free swimming poses reduces the network’s performance slightly (pose prediction score = 0.90), but illustrates that even free swims sample the gamut of poses needed to represent other behavioral contexts with high quality. As expected, this training dataset does perform better when modeling free swimming data only than when modeling the acoustic startle and dark flash data (**S8 Fig**). The network model trained on our two-camera dataset has a higher score compared to the three-camera dataset (see **S2 Table**). The use of a third camera view for 3-D reconstruction adds an overdetermined constraint, decreasing the prediction score, but increasing the quality of the estimate of the 3-D coordinates from 2-D projections.

### Physical model-trained convolutional neural network model is generalizable to novel data

While our pose estimation approach so far has been applied to recordings collected using our own 3-camera imaging system, we also wanted to assess its generalizability and robustness by testing it on datasets obtained under different imaging and experimental conditions. To this end, we analyzed sample recordings of larval swimming from other research groups, consisting of one 3-D dataset collected with a 2-camera system [37] and several 2-D datasets collected with a single overhead camera [37–39]. Three sets include prey capture [37,39], a behavior not present in our original training dataset. As a comparison to the external 3-D dataset, we also analyzed our data using only 2 of the 3 camera recordings (bottom and side cameras 1 and 2).

To analyze the literature 3-D data, we used the same ensemble of physical model poses as described above (represented in **Fig 1C and S2D Fig**), but created a new training dataset (**S2F Fig**) based on the different camera parameters used to make the recordings. Modeling the external 2-D datasets was more involved as it required the generation of a new ensemble of physical model poses. The approach was similar to the one used for our 3-D workflow. We obtained an ensemble of real poses from 2-D data of larvae swimming in a petri dish, collected by us with a 1-camera system [23], and used template-based optimization to determine the corresponding physical model parameters (**S1 Fig**). We set all adjustable parameters relevant to 3-D poses only, i.e. *φ*_0_, γ_0,_ *φ*_i_ and *z*_0_, to 0. We then performed uniform sampling to obtain an ensemble of 2-D physical model poses, where fish shapes having different mean backbone curvature <|Δ*θ*_i_|> were approximately equally represented. For computational efficiency, physical model images were rendered using orthographic projections of the voxel-based physical model (see **Materials and Methods: (b) Template-based pose estimation:** *Rendering larval projections*, *Physical model of the larva*). The training and validation dataset was obtained by splitting a set of annotated 500,000 rendered physical model images into a ratio of 9:1 respectively. We used this common trained model to perform pose estimation on all real 2-D datasets (see **Materials and Methods: (d) Modeling external data**).

**Fig 3:**
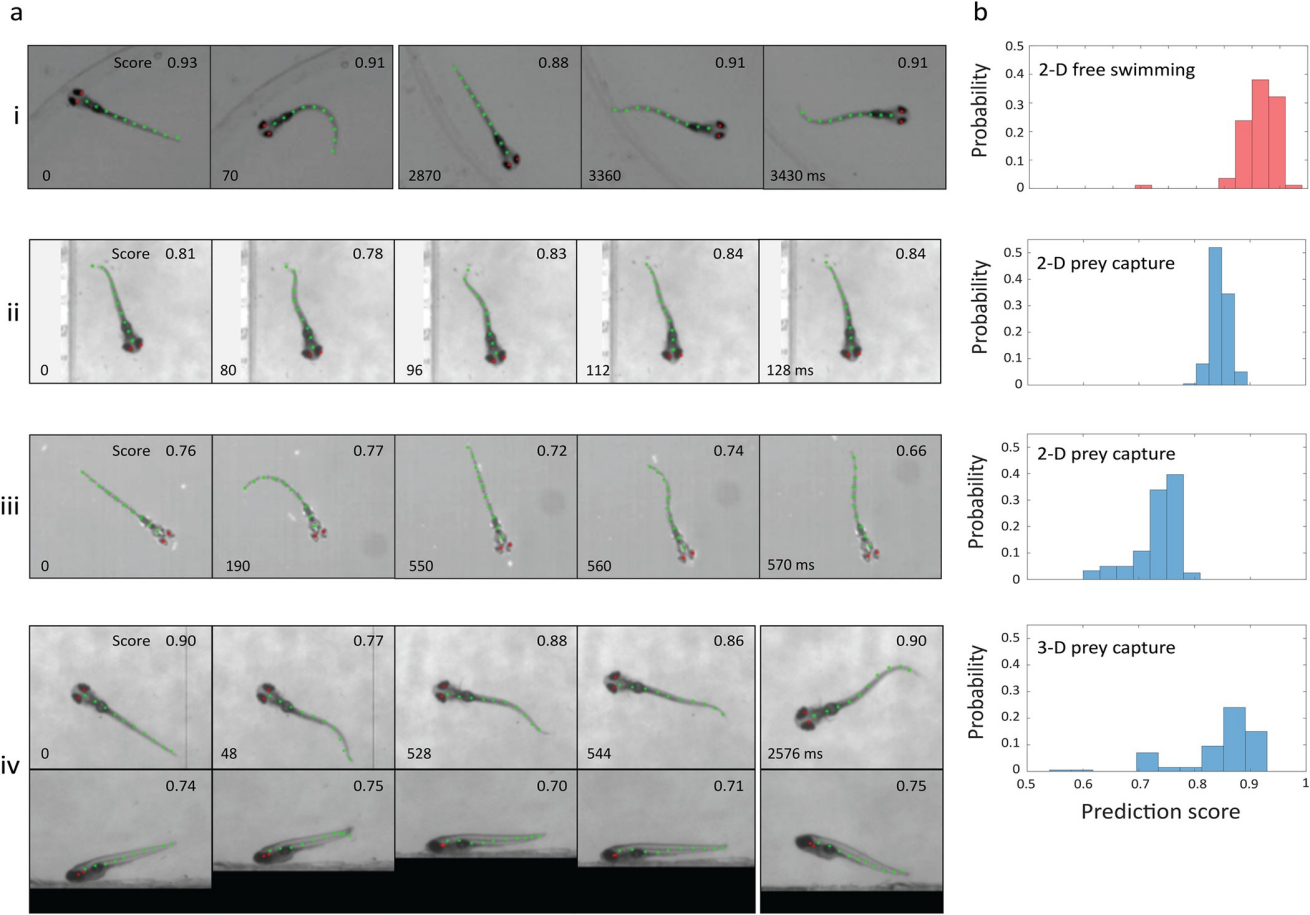
Physical model-trained neural network generalizes to external datasets in novel behavioral contexts. (a) Example sequences of raw images from datasets collected with other imaging systems, and superimposed 2-D projected poses from our neural network: ten 2-D backbone coordinates (green) and two 2-D centroids of eyes (red). Sampled frames are displayed for: (i) Multiple 2-D free swimming swim bouts from Ref. [38,40] showing diverse larval poses. (ii) A 2-D prey capture swim bout from Ref. [37], with the larva approaching a paramecium at 0 ms and successfully capture its prey over the next 128 ms. (iii) Multiple swim bouts from Ref. [39] showing a successful capture of a paramecium over the last three frames. (iv) 3-D prey capture swim bouts captured using a 2-camera setup from Ref. [37] showing a larva hunting a paramecium. (b) Pose prediction scores for all frames of the swim bouts are to the right of each example. The shift between pose prediction scores for different datasets reflects differences between imaging conditions and our physical model rendering.

**Fig 3** shows the pose predictions obtained on 3-D and 2-D external datasets. Selected movie frames from each dataset are displayed, with the coordinates of the physical model superimposed (**Fig 3A**). Despite the different imaging conditions (2-camera, 3-D imaging and 1-camera, 2-D imaging) and the novel behavioral context (prey capture) in these external datasets, visual inspection shows that our approach gives accurate pose predictions. **Fig 3A** shows the prediction scores corresponding to each frame, and **Fig 3B** displays the distribution of prediction scores from each datasets. Differences in the distributions across datasets reflect the different appearance of the larvae in the various recordings (**Fig 3A**) compared to those of the physical model images we optimized for our imaging system. We expect that re-training the network on physical model images designed to match those in the experimental data (see **Materials and Methods: (b) Template-based pose estimation**: *Physical model of the larva*) will increase the prediction scores, but emphasize that the pose prediction remains very accurate as it is, so improving the physical model imagery is not critical for good performance. We also show the prediction scores for our own dataset using 2 of the 3 camera images as a comparison to the external 2-camera 3-D dataset (**S9 Fig**). Our analysis demonstrates the robustness of the method to different types of recordings and behavioral contexts.

### 3-D kinematic parameters reveal unique features of larval swims across the three experiments

**Fig 4:**
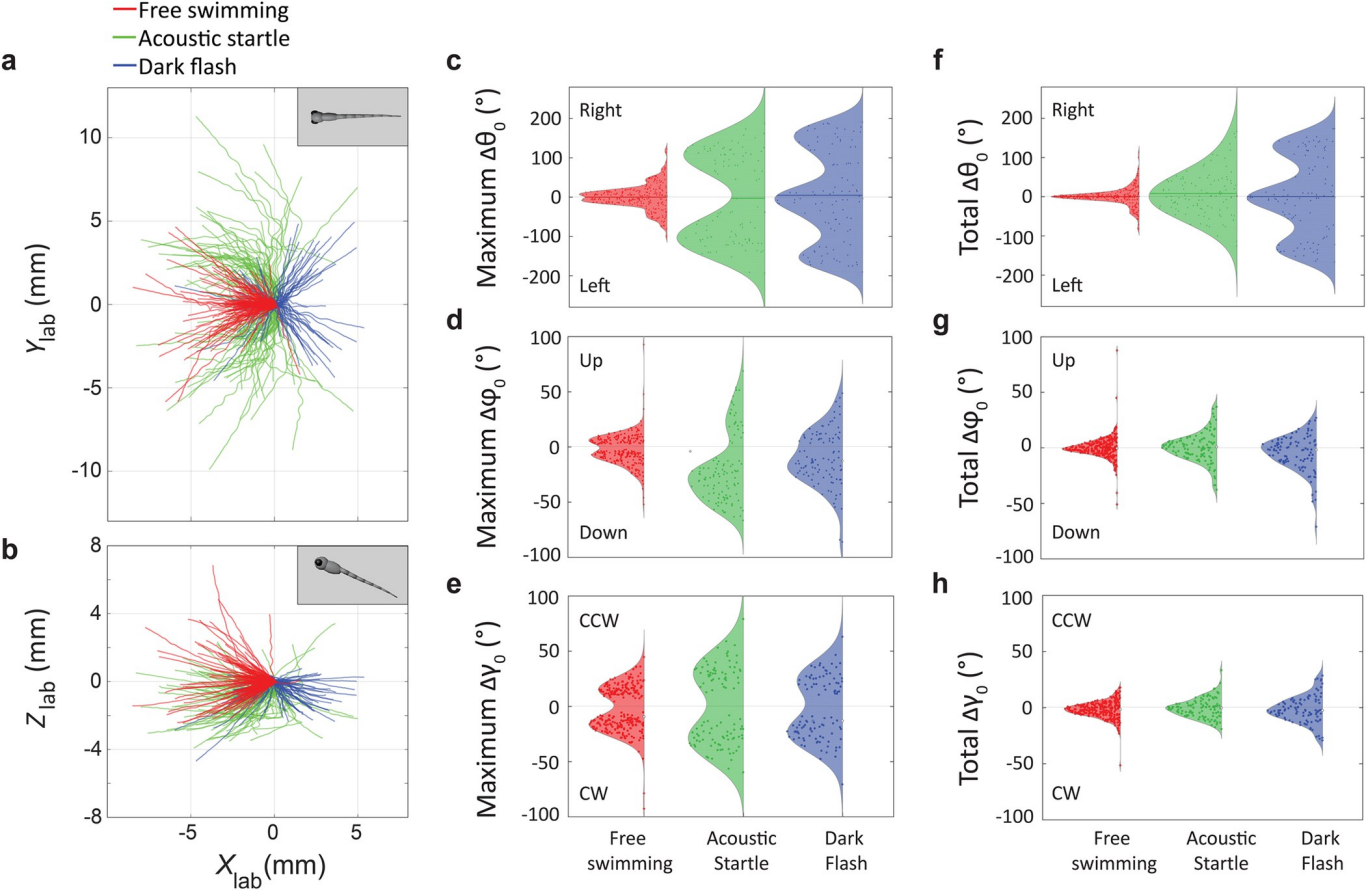
3-D kinematic parameters reveal unique features of larval swims across the three experiments. (a) Trajectories of the center of mass of larvae swimming in a 3-D environment (*N* = 478) in the *x*_lab_-*y*_lab_ plane, in all three experimental contexts (free swimming: red, acoustic startle: green, and dark flash: blue). (b) 3-D trajectories show a significant *z*-component, larger than the depth of ≈2 mm in constrained 2-D setups. (c-e) Distributions of the maximum change in the yaw *θ*_0_ (c), inclination angle *φ*_0_ (d), and roll angle *γ*_0_ (e) over a swim bout. Escape responses evoked by acoustic startles (green) involve the largest component of these motions. The asymmetry in the distributions of max(Δ*φ*_0_) of acoustic startle and dark flash responses shows the propensity of the larvae to dive in these experiments. (f-h) Distributions of total change in the yaw *θ*_0_, inclination angle *φ*_0_, and roll *γ*_0_ over a swim bout. The distributions in all three experiments peak at 0° showing the propensity of the larvae to reorient themselves to the pre-bout orientation by the end of the bout. The multimodal distribution of total change in *θ*_0_ for dark flash swim bouts (blue) captures the routine and strong turning maneuvers exhibited in that experiment.

We use the network predictions to inspect swimming parameters in the 3-D trajectories of larval swims. We performed pose prediction using the neural network over all the video recordings, comprising 630 swim bouts. We rejected any swim bout where at least one frame has a pose prediction score smaller than 0.85, resulting in 476 swim bouts (free swimming– 264, acoustic startle 109, acoustic startle– 103). **Fig 4A and 4B** shows the center of mass trajectories (*x*_0_, *y*_0_, *z*_0_) of the larvae recorded in free swimming, acoustic startle, and dark flash experiments. We find that these trajectories are symmetric in the *x*_lab_*-y*_lab_ plane. Thus, we rotated each trace along the *z*_lab_–axis such that the initial headings in the *x*_lab_*-y*_lab_ plane, *θ*_0_(*t =* 0), are oriented along the -*x*_lab_ direction. As seen in **Fig 4B**, the vertical motion of the larva has a significant component, with z-displacements several times larger in magnitude than the larva’s body length and the 2–3 mm height typically provided in constrained 2-D experiments [6,21,23]. During free swimming (red), vertical displacements span a range from -0.13 mm to 0.11 mm (lower and upper quartile throughout), mainly characterized by ascents. In contrast, in acoustic startle (green) and dark flash experiments (blue), vertical displacements range from -1.58 to 0.01 mm and from -0.83 to 0.01 mm, respectively, and are mainly characterized by descents. All differences in vertical range, not obtainable from 2-D experiments, are statistically significant and thus characteristic of different behaviors (p < 0.001, see **S3 Table**).

We also analyzed the yaw (*θ*_0_), inclination (*φ*_0_), and roll angles (*γ*_0_) of the larvae, the latter two parameters inaccessible in 2-D experiments. To quantify the variation in these parameters, we determined the interquartile range (IQR) of angles in these distributions. We find that *φ*_0_ varies between -14° and 10° (lower and upper quartile throughout) in free swimming experiments, -15° and 8° in acoustic startle experiments and -22° and 2° in dark flash experiments. *γ*_0_ varies between -5° and 5° for free swimming experiments, acoustic startle experiments, and in dark flash experiments. **Fig 4C–4E** show the distribution of maximum change in yaw max(Δ*θ*_0_), inclination max(Δ*φ*_0_), and roll max(Δ*γ*_0_) in a swim bout. We find that max(Δ*φ*_0_) varies in the range of -9° to 6° in free swimming experiments, -43° to 11° in acoustic startle experiments and -24° to 7° in a dark flash experiment. The lower and upper quartiles between free swims and startle/flash experiments are statistically significant. However, acoustic startle and dark flash experiments cannot be distinguished based on most of the angular ranges with the notable exception of the lower quartile of max(Δ*φ*_0_), suggesting larger dives for acoustic startle swim bouts compared to those from dark flash experiments (see **S3 Table**; **Materials and Methods: (e) Statistical Analysis**). The asymmetry between negative and positive max(Δ*φ*_0_) (**Fig 4D**) for acoustic startle (p = 6×10^-9^) and dark flash trajectories (p = 2×10^-3^) is consistent with the preponderance of dives in those experiments. On the other hand, the corresponding distribution of roll angles max(Δ*γ*_0_) is roughly symmetric, with the larvae exhibiting a larger range of rolls in response to acoustic startle stimuli (lower and upper quartiles for free swimming: -17° to 14°, acoustic startle: -30° to 30°, dark flash: -25° to 23°; again, only differences for free swims to other maneuvers are statistically significant (see **S3 Table** and see **Fig 4E** and **Materials and Methods: (e) Statistical analysis**). The distributions of total change (final–initial) in yaw Δ*θ*_0_, inclination Δ*φ*_0_ and roll Δ*γ*_0_ over a swim bout are shown in **Fig 4F–4H**. The distributions all have a maximum at 0°, which indicates that larvae have a propensity to reorient themselves and return to their initial heading in a majority of swim bouts. Despite the large lateral angle turns exhibited by larvae in acoustic startle experiments (see bimodal distribution of max(Δ*θ*_0_) in **Fig 4C**), the fish tend to return to a consistent ‘homing’ yaw (**Fig 4F**). Interestingly, the distribution of total Δ*θ*_0_ for dark flash swim bouts is multimodal, presumably capturing routine and strong turning maneuvers (i.e. O-bends), behaviors previously reported in 2-D assays [6,24,29,41].

**Fig 5:**
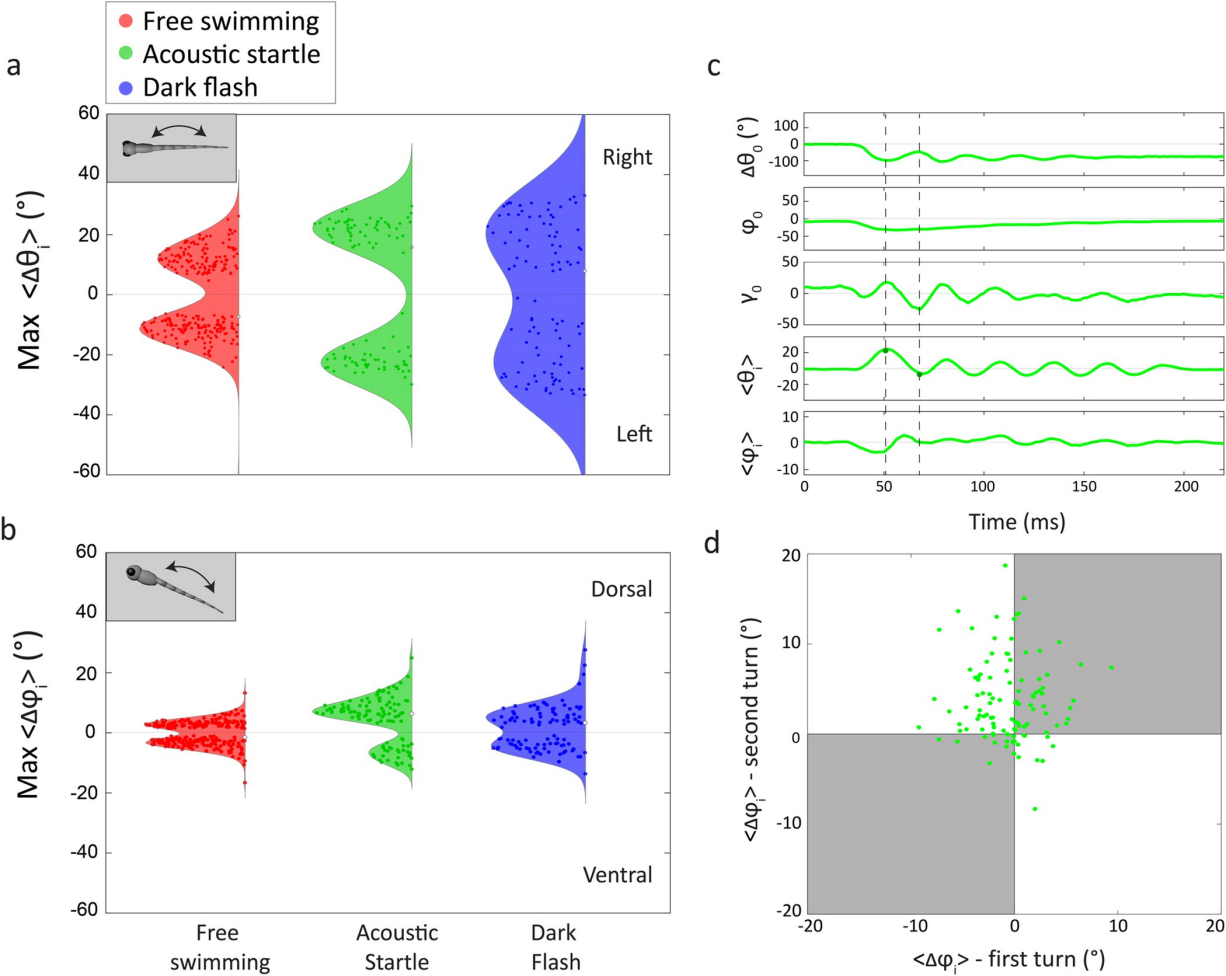
The larval body curvature correlates with 3-D swimming maneuvers. (a) Distributions of the maximum change in the average lateral bending angle <Δ*θ*_*i*_> over a swim bout in free swimming, acoustic startle, and dark flash experiments. Dark flash experiments elicit the largest lateral bending. (b) Distributions of the maximum change in the average dorso-ventral bending angle <Δ*φ*_*i*_> over a swim bout in the three experiments. Larva show an equal propensity for dorsal (positive <Δ*φ*_*i*_>) and ventral (negative <Δ*φ*_*i*_>) bending in free swimming and dark flash experiments, but a higher propensity for dorsal bends in acoustic startle experiments. (c) Representative time trace of the larval orientation and curvature from an acoustic startle experiment. A larva initiates the first turn (first dashed line, <Δ*θ*_*i*_>) with a ventral bend (<Δ*φ*_*i*_>), starting a dive (Δ*φ*_0_). The dive is arrested in the second turn (second dashed line, <Δ*θ*_*i*_>) by a compensatory dorsal bend. (d) Plot of the dorso-ventral bending amplitude in the first turn vs. the second turn in acoustic startle experiments. The predominant behavior consists of a first turn ventral bend followed by a second turn dorsal bend, corresponding to a diving maneuver.

As shown in **Fig 5**, our 3-D analysis allows us to go beyond the center-of-mass parameters *x*_0_, *y*_0_, *z*_0_, *θ*_0_, *φ*_0_, and *γ*_0_, and quantify parameters defining the 3-D shape of the larval backbone, *θ*_i_ and *φ*_i_. **Fig 5A** shows the distributions in the maximum change in lateral and dorsal bending angles, max(<Δ*θ*_i_>) and max(<Δ*φ*_i_>), occurring in each swim bout. These distributions show that larvae swimming in 3-D exhibit the highest lateral bending in dark flash experiments (-26° to 22°), followed by acoustic startle experiments (-23° to 23°), and by free swimming experiments (-11° to 11°). This trend is consistent with previous observations in 2-D experiments [5,6]. **Fig 5B** shows that the larvae also exhibit a considerable dorso-ventral bending in all the three experiments. The highest range of dorso-ventral bending is found in acoustic startle experiments (-9° to 4°), followed by dark flash experiments (-6° to 4°), and lastly by free swimming experiments (-3° to 3°). All differences in the lower and upper quartiles of max(<Δ*θ*_i_>) and max(<Δ*φ*_i_>) relative to free swims are statistically significant, and differences in lower quartiles are more significant for startle/flash than differences in their upper quartiles (see **S3 Table**). The asymmetry displayed for dorsal-ventral bending in acoustic startles is significant (p < 0.001).

**Fig 5B** shows that larval swim bouts in acoustic startle experiments generally display larger dorsal bending (positive <Δ*φ*_i_>), which would be expected to cause an increase in inclination *φ*_0_, whereas ventral bending (negative <Δ*φ*_i_>) would cause a decrease in *φ*_0_. This result is counterintuitive to our discussion of **Fig 4D**, which shows that acoustic startle swim bouts are mainly characterized by dives (i.e. decreasing *φ*_0_). Our ability to track the orientation angles and 3-D curvature of the fish in real time allow us to resolve this apparent conflict. **Fig 5C** shows an example time series trajectory of the larval orientation angles *θ*_0_, *φ*_0_, *γ*_0_, and curvature <Δ*θ*_i_> and <Δ*φ*_i_> for an acoustic startle swim bout. (The short response time <2 ms indicates that the bout is a short-latency C-start or SLC.) The first and second turns, time points in the bout where maximum bending generally occurs, are marked with dashed lines for visual reference. In this representative bout, the first turn is characterized by a ventral bend and a dive (see <Δ*φ*_i_> and *φ*_0_), followed by an equally sized dorsal bend into the second turn that stops the dive. An analysis of all acoustic startle bouts reveals a consistent pattern. **Fig 5D**, which plots the dorso-ventral bending amplitude in the first turn vs. the second turn, shows that the predominant behavior (42%; p = 10^−5^ for binomial test with 25% probability) consists of a first turn ventral bend followed by a second turn dorsal bend (see second quadrant in **Fig 5D**). Moreover, for those swim bouts the dorsal bend on average has a larger amplitude than the initial ventral bend (3.4° vs -2.7°). This finding indicates that acoustic startle dive maneuvers consist mainly of a first turn ventral bend followed by a compensatory dorsal bend that terminates the dive (similar to the representative trajectory in **Fig 5A**). On average this dorsal bend represents the largest bend during the bout, leading to the observed asymmetry in the dorso-ventral bending distribution in **Fig 5B**. (We note that for acoustic startle swim bouts that initiate with a dorsal bend in the first turn, the second turn also mainly (80%; p = 5×10^-6^ for binomial test with 50% probability) consists of a dorsal bend.) Our result paints a more subtle picture of the interaction between epaxial and hypaxial muscles to initiate a ’dive and break,’ as also investigated in another 3-D neurokinematic study [27]: a dorsal bend following the ventral bend that initiates the dive has enhanced probability to prevent over-pitching of the body. The ability of our 3-D pose prediction method to determine accurately all larval posture angles simultaneously, at high throughput, allows us to identify previously unreported yet statistically significant swimming maneuvers in 3-D contexts. Moreover, the significance of dorso-ventral bending motion in our 3-D dataset can be seen by performing Singular Value Decomposition (see Ref. [23] for detailed analysis) on **ϴ**(*t*), which reveals significant orthonormal bending modes comprising mainly dorso-ventral bending (Δ*θ*_i_ ~ 0) motion (see **S10 Fig**).

## Discussion

The reconstruction of larval zebrafish swimming behavior in three dimensions has long been a challenge, with several obstacles such as the high speed and small size of the animal, refraction from water and glass, and the semi-transparent body of the larvae. While the latter trait facilitates imaging of its organs and tissues [25,42,43], it complicates the imaging of behavior because the posterior part of the tail is more difficult to distinguish from the background. In addition, the brightness of the fish image depends on its orientation relative to the camera because imaging systems record the light transmitted through the organism’s body. Here, we present a tracking algorithm based on a physical-model trained neural network analysis of fish videos that produces an accurate coordinate representation of its poses and behavior in 3-D using relatively low-resolution cameras (648 × 488 pixels) taken by an affordable multi-camera setup. Its robustness is illustrated by the quantitative evaluations of the network’s performance, and its ability to process external datasets accurately despite the different imaging and experimental conditions. Since the parameters of the projection function are not baked into the network model, the convolutional neural network model is agnostic to the 3-D to 2-D mapping for the imaging system used. We find that the network’s performance does not change due to small drifts in the cameras occurring over time. This is reflected in the fact that the projection function used to generate the training dataset was different from that calibrated for free swimming experiments. However, for significant deviations in the 3-D to 2-D mapping, a new training dataset does need to be generated, as done for the literature 2-camera 3-D dataset imaged using telecentric lenses [37] (**Fig 3**).

The approach of generating synthetic images to tackle several pattern recognition problems using a neural network has been used in the past [12,44–51]. It provides two advantages over collecting and manually annotating raw video data as training data: (1) generation of an arbitrarily large dataset to train artificial neural network models, and (2) accurate annotations of the dataset without needing human intervention. However, in many of these examples, there is the risk that features of the synthetic images are significantly different from those of real images, and a network trained on synthetic datasets does not provide acceptable results on real datasets. One approach to circumvent this problem is to use parts of real images to generate the synthetic training dataset [12,52]. Here, simple affine transforms (like scaling, rotation, translation) applied to ‘template’ images are used to generate new training examples. Such a use of real images plausibly preserves the relevant textural features of real images in the generated training dataset. However, the use of such image transforms is not practical in our 3-D context, where some part of the larva is always self-occluded on one or more camera views (an obvious example being one of the eyes). It is not possible to use affine transforms between two image frames that contain different parts of the fish due to occlusions. While self-occlusions are more prevalent for the side cameras, bottom cameras also face this problem for swim bouts containing significant vertical motion.

For this reason, we chose to render physical model images digitally using a physical model-based approach without explicit use of any real images. Our fully digital data seamlessly integrates into the training pipeline. In other examples in the literature [53,54], significant differences between digitally generated training datasets and real images have necessitated new approaches to integrate the digital datasets into the analysis pipeline. Research in the field of *domain adaptation* is an ongoing effort to address these challenges [47,55–58]. Interestingly and somewhat surprisingly, our physical model-trained convolutional neural network is able to perform accurate pose estimation on a diverse range of real 2-D and 3-D datasets collected with different imaging systems with no need for domain adaptation. We speculate it this may be due to the variance in the fixed parameters of the physical model used to render the physical model images.

We anticipate that this rich and annotated digitally generated dataset can be used in many other applications. While our current network model only predicts the pose of a single larva, one such immediate application may be studying a group of larval zebrafish. Research groups have already had success tracking multiple animals using artificial neural networks [59–61] that rely on good training datasets. Our physical model approach allows us to render multiple fish in a single image and track them (**S11 Fig**). A dataset of a group of larval fish generated using the physical model could be used to train a network inspired by one of these studies, by using a suitable loss function compatible with different numbers of animals in different video frames. A model trained on such a dataset should be able to predict poses of multiple larvae simultaneously. This may open avenues to questions about the social behavior of zebrafish larvae freely swimming in a 3-D environment. Our fully annotated larval zebrafish dataset can also serve as a benchmark for upcoming tools aimed not only at animal pose estimation, but more generally to computer vision problems like 3-D reconstruction and pose estimation.

We also anticipate that with higher resolution cameras, our system could in the future allow the study of pectoral fins, movement of eyes [62], and other attributes. These features can easily be incorporated into the physical model by adding a few additional coordinates. Notably, the eyes in our physical model are already parameterized by their orientation, although this was constrained to be fixed in the generation of our physical model images. Extending our method for studying such attributes would require generating an ensemble of realistic poses that include fins, eyes, and other parts of the organism and knowledge of any correlations in their motion. We expect that a network trained on such a dataset would be capable of modeling pectoral fin and eye movements, which have been proposed to play roles in behavior [63–66].

Our dataset of temporally independent poses may also be used to generate datasets of temporally correlated sequences of poses, as exhibited in stereotyped behaviors. Such datasets may be useful to learn an algorithm that can predict the future poses of a larvae, given a sequence of poses in the past. Such an algorithm could be useful in improving hardware that requires online tracking of fast swimming larvae [25] for simultaneous behavioral/neuronal recordings. The use of a generative model that learns temporal relationships in the data may help create such a dataset. Such a model may be trained by using a powerful neural network model [67]. One could also use a more physics-based approach by learning the postural dynamics using closed-form expressions. An example of such an approach is a recently published work by [4], where high-dimensional postural dynamics of a moving worm were approximated as a series of linear stochastic differential equations, inferred from the data.

By marrying neural network learning with physically constrained synthetic training datasets, our approach allows tracking 3-D dynamics of larval poses with a detail and throughput that was not previously achieveable. We show that larval dynamics, especially in acoustic startle and dark flash experiments involve a notable vertical component. Moreover, larvae also exhibit interesting dorso-ventral dynamics, which could not have been analyzed at a sufficiently high throughput for statistically significant numbers without an efficient and automated tool such as ours. Thus, we anticipate that our dataset of 3-D larval poses will rouse interest among ethologists studying zebrafish larvae to investigate novel aspects of their 3-D poses and dynamics, previously unexplored in constrained 2-D experiments. Furthermore, our automated method allows for high throughput analysis of 2-D imaging datasets, which are increasingly becoming larger in size. We hope that our system significantly reduces the challenges for zebrafish researchers to perform more behavioral experiments with zebrafish larvae in more native-like environments.

## Materials and methods

### Ethics statement

All behavioral experiments were performed on larvae between 6 and 9 dpf in accordance with the protocol (#19100) approved by the Illinois Institutional Animal Care and Use Committee.

#### (a) Instrumentation

*Animals*. Adult AB genotype zebrafish larvae were obtained from the Zebrafish International Resource Center (ZIRC, Oregon). Larvae used in the experiments were obtained by breeding the adult fish in the lab and raised at 28°C in Danieau’s solution [68] until 6 dpf (days post fertilization).

*Experimental setup and data collection*. The experimental setups used for the 3-D experiments are shown in **S3 Fig**. Fish swimming measurements in 3-D were carried out in a custom-built cubic glass tank of dimensions 7 × 7 × 7 cm. Zebrafish larvae at 6–9 dpf and measuring a few mm in length were placed in the tank and were allowed to swim freely without external stimulus. Movies of the larvae were obtained at 500 fps using 3 synchronized high-speed cameras (Ximea xiQ MQ003MG-CM) aligned with their principal axes orthogonal to each other and intersecting at a unique point in the center of the tank. The cameras were synchronized with a waveform generator (33250A, Agilent), which generated a 500-Hz square wave to trigger the three cameras at 2-ms intervals with 0.1-μs timing tolerance. The cameras were aligned as follows. Briefly, a mirror was placed facing the camera to be aligned, with its surface along the nearest face of the cubic tank. The center of the camera lens seen in its reflected image was detected using image analysis. In order to align the principal axis perpendicular to the mirror (and hence to the face of the tank), the (detected) center of the lens was made to coincide with the center of the image by rotating the camera on its mount. Similarly, the other two cameras were also rotated. To ensure that their principal axes intersected at the center of the tank, we translated the cameras using the detected centers as reference. Infrared illumination at wavelengths >700 nm enabled the same imaging modality for all experiments, including dark flashes. We used 3 orthogonally placed and diffused infrared lamps (Chanzon, λ = 850 nm) with a high-power output (50 W) to compensate for the high frame rate and small optical aperture (large *f*-ratio) of the cameras, which was necessary in order to achieve a large depth of field.

Zebrafish larvae were habituated in the 3-D arena for 10 minutes after being transferred from the incubator. Movies of larval swimming were then collected in three experimental contexts: free swimming, acoustic startle, and dark flash stimulus. Acoustic startles were generated by releasing a small cylindrical weight from an electromagnet on the platform supporting the glass cube (**S3C Fig**). Vibrations generated in the tank from the impact of the weight on the platform were minimal (<70 μm, estimated from imaging of the dot grid target; see **Materials and Methods: (a) Instrumentation:** *Camera calibration*: *Mapping from lab frame to camera coordinates*). A dark flash stimulus was provided by initially habituating the larva to a white light LED for at least 4 minutes before instantaneously turning the light off for 10 s (**S3B Fig**). Automated stimuli were generated using an Arduino UNO board interfaced with MATLAB and the stimulus source (electromagnet/white LED) triggered using an NPN transistor.

It was rare for a larva to be in the field of view of all three cameras at the same time. Thus, a search for such a larva was conducted once every second and a stimulus was triggered only when one was detected simultaneously by all the three cameras. Since the camera parameters that project a real 3-D point in the 2-D pixel space are non-linear, triangulating the position of the larva using the center of mass detected by the cameras required non-linear optimization. This process is computationally expensive in MATLAB and prevents monitoring the larvae in real time, once a second. Thus, we used the following algorithm to perform this real-time search:

1. Detect the centroids {*C*_⍺_^*i*^}, where *C*_⍺_^*i*^ ∈ ℝ^2^ is the centroid of the *i*^th^ larval projection on camera α, where α ∈ {1,2,3} and *i* ∈ {1,2,3,..,*N*} and *N* is the total number of larval projections detected in camera α’s field of view.
2. Determine *R*_⍺_^*i*^, the ray of light incident on pixel *C*_⍺_^*i*^ of the camera sensor array, using Lookup Table R (see **Materials and Methods: (a) Instrumentation:** *Camera calibration*: *Reverse mapping from camera coordinates to lab frame via Lookup Table R*). By construction of the lookup table, this ray of light should have originated from a point coinciding with a larva in the tank.
3. Determine all the tuples (*R*_1_^*i*^, *R*_2_^*j*^, *R*_3_^*k*^) iterating over all *i*, *j*, *k* in the three cameras.
4. For each tuple (*R*_1_^*i*^, *R*_2_^*j*^, *R*_3_^*k*^), compute δ = max[*d*(*R*_1_^*i*^,*R*_2_^*j*^), *d*(*R*_2_^*i*^,*R*_3_^*k*^), *d*(*R*_1_^*j*^,*R*_3_^*k*^)], where *d*(*R*_*α*_^*m*^,*R*_*β*_^*n*^) is the minimum distance between the rays projecting on the pixel *C*_⍺_^*m*^ of camera α and *C*_β_^*n*^ of camera β.
5. If δ < ε, the rays (*R*_1_^*i*^, *R*_2_^*j*^, *R*_3_^*k*^) originate from the same larva (ε is a small positive number determined heuristically).

We also performed 2-D behavioral experiments to develop a 2-D pose estimation workflow (see **Materials and Methods(d) Modeling external datasets:** *2-D pose estimation*: *Model training*). We collected free swimming, acoustic startle and dark flash videos using the imaging setup described in Ref. [23]. Acoustic startles were generated using a speaker (40 Hz monotonic signal designed using MATLAB) and dark flash experiments were performed by habituating the larvae in white light for 4 minutes and suddenly turning the white LED off, using an Arduino UNO board. We collected a total of 289 videos (free swimming: 88 acoustic startle: 82, dark flash: 119), each consisting of multiple swim bouts (see **Materials and Methods: (b) Template-based pose estimation:** *Optimization*).

*Camera calibration*: *Mapping from lab frame to camera coordinates*. In the absence of the water tank, the mapping from a 3-D lab frame coordinate (*x*_lab_, *y*_lab_, *z*_lab_) to a 2-D point on the camera frame (*x*_*i*_, *y*_*i*_) is a straightforward linear operation given the camera matrix $P_{i},\mathrm{via}\;\mathrm{the}\;\mathrm{formula}[x_{i}{,y}_{i},1]=[x_{lab}{,y}_{lab},z_{lab},1]P_{i}$, where *i =* {1,2,3} is the index of the camera [69]. The camera matrix **P**_*i*_ is a 4 × 3 matrix modeled as a product **M**_*i*_ **K**_*i*_ of a 4 × 3 ‘extrinsic’ matrix **M**_*i*_ and a 3 × 3 ‘intrinsic matrix’ **K**_*i*_. **M**_*i*_ is determined by extrinsic parameters characterizing the rotation (quantified by the Euler rotation matrix, *R*_3x3_) and translation (characterized by the vector *T*_3x1_) of each camera relative to the lab coordinate system. The ‘intrinsic’ matrix **K**_*i*_ is determined by intrinsic parameters of the camera such as the focal length (in pixels), *f*_*x*_, *f*_*y*_; principal point coordinates (in pixels), *μ*_0_ and *v*_0_; and the skew coefficient of the pixels *γ*. A dot grid target (R2L2S3P4, Thorlabs) was used in the camera calibration process to determine all the camera parameters listed in the matrices **M**_*i*_ and **K**_*i*_ (see **S1 Table**). Several sets of images of the target at different orientations to the camera were taken (shown in **S4A Fig**) for camera calibration. The positions of the dots were detected using a custom-written code. Camera calibration was performed with the Computer Vision System Toolbox in MATLAB.

In fish swimming measurements, the projection from a 3-D lab coordinate of a point on the fish to its 2-D coordinate on the camera is no longer a linear operation because of the refraction caused by water and glass. There still exists a function *f*_*i*_ for camera *i* such that (*x*_*i*_, *y*_*i*_) = *f*_*i*_(*x*_*lab*_, *y*_*lab*_, *z*_*lab*_). However, an accurate analytical expression for this function is cumbersome and slow to compute. Rather than seeking the exact solution, we obtained an empirical function *f*_*i*_ by fitting images of the dot grid target. First, we collected a set of pictures of the dot grid target without the cubic tank (**S4A Fig**). Then, we placed the filled tank around the target while keeping the target at the same position as before and collected another set of pictures (**S4B Fig**). Replacing the tank surrounding the grid required removing the grid from its position. A kinematic base (KB50/M, Thorlabs) allowed us to position the grid in the same position and orientation after placing the tank. We obtained two sets of 2-D projection coordinates of the dot patterns, $\left\{r_{ni}^{water}\right\}$ and $\left\{r_{ni}^{air}\right\}$ for each camera, corresponding to the images taken with and without refraction due to the tank and water, respectively. Here, $r_{ni}^{water}$ = (*x*_*ni*_, *y*_*ni*_) are the 2-D projection coordinates of dot *n* ∈ {1,2,3,..,*N*} on the camera *i =* {1,2,3} in the presence of refraction due to the tank and water, and $r_{ni}^{air}$ are the 2-D projection coordinates of the same dot *n* on the same camera *i* in the absence of refraction (**S4C Fig**). The 3-D lab coordinates of the *N* dots $\left\{R_{ni}^{air}\right\}$ were then reconstructed from $\left\{r_{ni}^{air}\right\}$, by minimizing the function $\sum_{n=1}^{N}\sum_{i=1}^{3}{\left|(r_{ni}^{air}-P_{i}{R_{n}}^{air})\right|}^{2}$. Because the lab coordinate system *x*_*lab*_, *y*_*lab*_, *z*_*lab*_ in **S1C Fig** is based on one of the cameras, with three cameras perpendicular to each other, we found that it was possible to simplify the projection function by calculating the two camera coordinates (*x*_*i*_, *y*_*i*_) independently. For instance, for camera 1, in the relation (*x*_1_, *y*_1_) = *f*_1_(*x*_*lab*_, *y*_*lab*_, *z*_*lab*_), with *x*_1_ being perpendicular to *y*_*lab*_, the value of *x*_1_ was independent of *y*_*lab*_. Thus, the projection function could be separated into two independent functions *x*_1_ = *f*_1*x*_(*y*_*lab*_, *z*_*lab*_) and *y*_1_ = *f*_1*y*_(*x*_*lab*_, *z*_*lab*_), each of which was fitted to a cubic function for interpolation to achieve satisfactory accuracy. As shown in **S4D Fig**, the fit function *f*_1*x*_ matched the experimental data (plotted as gray dots) well. With the empirical projection functions, we are able to reduce the mean triangulation error from 1.07 mm to 30 μm (**S4E Fig**), a small fraction of the body diameter of the animal.

*Camera calibration*: *Reverse mapping from camera coordinates to lab frame via Lookup Table R*. Having found the mapping from 3-D lab frame coordinate (*x*_lab_, *y*_lab_, *z*_lab_) to 2-D point on the camera frame (*x*_*i*_, *y*_*i*_), it is necessary for analyzing larval swimming movies to construct the reverse mapping from (*x*_*i*_, *y*_*i*_) to the set of 3-D coordinates that project onto (*x*_*i*_, *y*_*i*_). Given that the laws of ray optics hold true for our setup, we expect a set of coordinates on a unique ray, **a** + Λ**r**, to be projected, after refraction through the water-glass-air interface, onto a given pixel (*x*_*i*_, *y*_*i*_) of the sensor array of camera *i* (**S5 Fig**). Here, Λ parametrizes the ray, **a** ∈ *R*^3^ defines the origin of the ray in the lab reference frame at Λ = 0 and **r** ∈ *R*^3^ is the directional vector of the ray, defining the slopes along the 3 lab coordinate axes. The origin of the ray is defined as the point of intersection of the ray with the plane perpendicular to the principal axis of the camera, passing through the center of the tank. Since determining the set of 3-D points whose projection falls on the pixel (*x*_*i*_, *y*_*i*_) involves nonlinear optimization, it is computationally inefficient and thus motivates the use of a lookup table. For each camera, an independent ‘Lookup Table R’ for aiding in reverse mapping stores the vectors **a** and **r** for every pixel (*x*_*i*_, *y*_*i*_) of the camera sensor array. The values of **a** and **r** are determined by finding three random 3-D points in the tank that are projected onto the given pixel (*x*_*i*_, *y*_*i*_), using the non-linear mapping functions *f*_*i*_(*x*_*lab*_, *y*_*lab*_, *z*_*lab*_) above, and fitting a line through these three points. Lookup Table R is used in triangulating the center of mass of larvae in the tank (see pseudo code in **Materials and Methods: (a) Instrumentation:** *Experimental Setup and Data Collection* above) and in fitting the three camera images to a physical model of the larva (see **Materials and Methods: (b) Template-based pose estimation:** *Physical Model of the Larva*).

#### (b) Template-based pose estimation

*Preprocessing*. The videos of larval swimming were preprocessed to improve the speed and accuracy of larval pose estimates. The complete preprocessing pipeline is described in (**S7 Fig**). The raw images were background-subtracted to increase the contrast between the foreground and the background and to reduce noise. The background image was created by computing the 90^th^ percentile intensity of every pixel over the entire video. This image was then passed through a Gaussian filter (window size: 5 × 5 pixels, standard deviation: σ = 1) to reduce high frequency noise further. The images of the larva were then segmented into binary images using a heuristically defined intensity threshold, and a bounding box was defined around the larva. The size of the bounding box was determined so that the largest 2-D projection of the larva (seen when the larva is closest to the camera) could be contained within the box. Since multiple projections of larvae were often detected in several frames, the larva of interest needed to be tracked. Tracking over multiple frames was performed by triangulating the centroid of the larva in 3-D coordinates in the current frame and detecting the nearest neighbor centroid in the following frame. For all the analysis downstream, we used this background subtracted and cropped image. Note that all images illustrated in the figures are these preprocessed images.

*Physical model of the larva*. We defined a physical model of the larva using 22 adjustable parameters (**Fig 1C**) and 20 fixed parameters (see below). We showed previously that 10 segments are sufficient to characterize the variations in the shape of the larval backbone [23]. Using 10 segments also allows for direct comparison of our results with the previous 2-D data [23]. The model is based on a scaffold comprising a chain of 9 rigid segments, allowing for 2 degrees of freedom for every joint connecting successive pairs of segments. Thus, the larval pose, representing the shape of its backbone, is defined in our framework by 16 parameters Δ*θ*_1_,… Δ*θ*_8_, Δ*φ*_1_,… Δ*φ*_8_ that represent the radial and azimuthal angles of each segment measured relative to the larval head orientation. Our choice of the number of segments defining the physical model is constrained only by computational power and the imaging resolution. In principle, the entire analysis can be reproduced for an arbitrarily large number of segments. The remaining six degrees of freedom—coordinates of the center of mass (*x*_0_, *y*_0_, *z*_0_) and the yaw *θ*_0_, pitch *φ*_0_, and roll *γ*_0_ of the larval head—establish the position and orientation of the larva in the lab reference frame, which is defined with respect to the bottom camera.

The anterior of the larva (eyes, head, and belly) is modeled as a set of ellipsoids, with the first rigid segment used as a scaffold (**S6A Fig**). We use the following 19 fixed parameters to define these parts of the larval anterior: brightness of the eyes, head, and belly (3 parameters); distance between the eyes (1 parameter); width, length, and height of the eyes, head, and belly (9 parameters); position of the eyes, head, and belly with respect to the scaffold segment (6 parameters). The position of the belly and head with respect to the scaffold segment can be defined uniquely using only two parameters each, since the belly and head have a constraint to be laterally symmetric about the scaffold segment. The position of the eyes is also defined using 2 parameters, because the centroids of the eyes are constrained on a segment perpendicular to the scaffold segment, with each centroid located at the end of the segment. The length of this segment is twice the distance between the eyes. For a given roll angle of the larva, this segment can be uniquely determined using two parameters: its perpendicular distance from the scaffold segment and its position along the scaffold’s length. The length of the larva *L* brings the count of fixed parameter to 20. *L* also serves to scale the absolute size of the eyes, head, and belly of the physical model of the larva during the second round of optimization (see **Materials and Methods: (b) Template-based pose estimation:** *Optimization*). A scaling parameter for the length of the larval anterior, *l*_*A*_, which depends on *L* and the position of the larva in the tank, is used for rendering 2-D projections of the anterior in the first round of optimization (see **Materials and Methods: (b) Template-based pose estimation:** *Optimization*).

The larval posterior (tail) segments are constructed on the scaffold of the remaining 8 rigid segments, with a single fixed parameter scaling the width of the tail segments. This parameter was determined by averaging images of multiple larvae. Since our results were found to be robust to small changes to the width of the tail segments, the scaling parameter for the width is determined uniquely for all swim bouts and kept fixed for the entire analysis. In addition, the length of each tail segment is set to 1/9^th^ times the length of the whole larva *L*, allowing it to vary between larvae.

The larval anterior (head, eyes, and belly) and the posterior (tail segments) are rendered independently. For the anterior, we used a voxel-based 3-D model to generate 2-D projections that recreate the preprocessed images (**S1F** and **S6A Figs**). The intensity profile *V*(*x*_*lab*_, *y*_*lab*_, *z*_*lab*_) of the voxels is given by max(**N**(*μ*_eye1_,Σ_eye1_), **N**(*μ*_eye2_,Σ_eye2_), **N**(*μ*_head_,Σ_head_), **N**(*μ*_belly_,Σ_belly_)), where **N**(*μ*,Σ) is the normal distribution with mean μ and covariance matrix Σ, uniquely defined by the fixed parameters. *μ*_k_ ∈ *R*^3^ is the center of mass of organ ‘*k’* and Σ_*k*_ is a 3 × 3 covariance matrix that encodes the dimensions of *k*. To obtain 2-D projections of the voxels, we either perform an orthographic projection or a nonlinear projection using the camera matrix, depending on the round of optimization (see ***Image Analysis***: *Optimization* and **S1D–S1F Fig**). Pixel intensities of the projections are obtained by summing over the intensity of all the voxels that are projected on the respective pixel. For example, for the orthographic projection with respect to the bottom camera (camera 1 in **S1 Fig**), pixel intensities *P*_zlab_(*x*_lab_,*y*_lab_) are given by ∑_*zlab*_*V*(*x*_*lab*_, *y*_*lab*_, *z*_*lab*_).

For the larval posterior, the 2-D projections of the tail segments are rendered using a voxel-equivalent model to make the optimization more efficient in time and memory. In this model, the projections of the tail are constructed on the scaffold of the 8 rigid segments using a series of trapeziums connected by disks (**S6B Fig**). The intensity of each of these segments has a Gaussian profile along the width and a linear profile along the length, decreasing in the belly-tail direction. This construction is equivalent to the projection of a voxel-based model consisting of tapered cylinders connected by spheres.

*Rendering larval projections*. We generate realistic larval projections using either of two approaches differing in computational complexity: (i) Using Lookup Table P to render larval projections completely, (ii) Using Lookup Table P to render the larval posterior (tail segments), while the larval anterior is rendered by projecting individual voxels of the voxel-based model (see **Materials and Methods: (b) Template-based pose estimation**: *Physical model of the larva*) using the projection function (see **Materials and Methods: (a) Instrumentation**: *Camera calibration*: *Mapping from lab to camera coordinates*). We employ (i) during the first round of optimization (see **Materials and Methods: (b) Template-based pose estimation**: *Optimization*), where it is critical to render larval projections rapidly. On the other hand, (ii) is used during the second round of optimization and while rendering physical model images used to train the neural network (see **Fig 1E**), where generating accurate projections is critical while sacrificing on the computational speed.

In order to construct larval projections from Lookup Table P for a given parameter vector **p** ∈ *R*^22^, along with *L*, we render the larval anterior and posterior independently. The procedure for generating digitized larval projections during optimization using Lookup Table P involves determining the appropriate lookup table indices corresponding to the scaling parameter and orientation of the larval anterior and tail segments. The procedure to render larval anterior using Lookup Table P is described in detail in **SI: Rendering larval anterior using Lookup Table P**).

For rendering the tail, this procedure is not necessary because the indices of the tail segments, by construction of Lookup Table P for tail segments, are defined with respect to the orientation, length, and offset of the 2-D projections explicitly. We first compute the 2-D projections of the larval backbone (the chain of 8 rigid segments forming the posterior) using the non-linear projection functions *f*_*i*_(*x*_*lab*_, *y*_*lab*_, *z*_*lab*_) described above (see **Materials and Methods: (a) Instrumentation**: *Camera calibration*: *Mapping from lab to camera coordinates*) and determine their projected length. This gives the index corresponding to the scaling parameter *l*_*T*_. The entry of Lookup Table P determining the orientation for the tail segments can be evaluated directly by computing the orientation of the 2-D projection of the individual tail segments forming the larval backbone. Lastly, the subpixel offsets can be determined by computing the fractional part of their *x* and *y* coordinates.

We also render the larval anterior directly using the voxel-based model in some cases, as described above in this section in (ii). Rendering every voxel individually using the non-linear projection function (see **Materials and Methods: (a) Instrumentation**: *Camera calibration*: *Mapping from lab to camera coordinates*) assures higher accuracy but is computationally expensive. While rendering the larval anterior in this way, a voxel-based model of the 3-D larval anterior is first generated (see **Materials and Methods: (b) Template based pose estimation**: *Physical model of the larva*). Every voxel is mapped from the 3-D lab-coordinates to the 2-D camera coordinates and the voxel intensities projected at every camera coordinate are summed, resulting in a 2-D image. Further, the intensity of the 2-D image is scaled such that the brightest pixel has a value of 255.

*Optimization*. The physical model of each larva is constructed from the unique chain defined by the parameter vector **p** ∈ R^22^: **p** = (*x*_0_, *y*_0_, *z*_0_, *θ*_0_, Δ*θ*_1_,… Δ*θ*_8_, *φ*_0_, Δ*φ*_1_,… Δ*φ*_8_, *γ*_0_), in addition to the total length *L* of the larva. In order to compute the larval pose in any frame of a swim bout movie, we perform a search over the space of 22 adjustable parameters such that digitally generated larval projection images of the physical model best match the three views recorded by the cameras. A cost function, defined as the sum of the 2-norms of the three difference images (of the background subtracted image and the digitally generated projection) for the three cameras, is minimized globally using the *patternsearch* algorithm in MATLAB’s Global Optimization Toolbox. Each frame of the bout is analyzed in parallel on a node of an Intel Xeon Gold 6150 @2.7 GHz cluster.

The entire pipeline for the analysis of swimming movies is described in **S1 Fig**. We performed the optimization of the physical model against the collected data in two rounds. In the first round, a coarse estimate of the model’s parameter vector **p** is generated using a lookup table (see **Materials and Methods: (b) Template-based pose estimation:** *Lookup Table P*) that stores a large array of larval projections (**S1E Fig**). In the second round, fine optimization improves the estimate in a small neighborhood of **p** obtained in the first round (**S1F Fig**). Here, the larval projections are obtained by rendering the voxels defining the physical model at every iteration (see **Materials and Methods: (b) Template-based pose estimation:** *Rendering larval projections*).

Before initializing the optimization, we estimate the fixed parameter *L* from the length of the binary, segmented image of the larva from the bottom camera in the initial and final 5 frames of the swim bout, during which the larval backbone is expected to be straight. Specifically, we first find the best orientation angles of the larva (*θ*_0_, *φ*_0_) that minimize the cost function, assuming a physical model with a straight backbone and a larval length of 4.5 mm. *L* is averaged over all 10 frames.

In the first iteration of the coarse optimization, the adjustable parameters are initialized by an approximate guess of the centroid of the larva (*x*_0_, *y*_0_, *z*_0_), triangulated using the centroids of the segmented larval images captured in the three cameras. This triangulation is performed using the nonlinear projection function by nonlinear optimization (*fmincon* function in MATLAB). Since recording is started before the stimulus is generated, we expect the larval backbone to be a straight line in the first frame and find the best orientation (*θ*_0_, *φ*_0_, *γ*_0_) that minimizes the cost function. For this step, we use a brute force approach by iterating over a large number of orientations (*θ*_0_, *φ*_0_, *γ*_0_) and render the corresponding digital projection of the larva (see **Materials and Methods: (b) Template-based pose estimation:** *Rendering larval projections*). The constraint on the shape of the backbone significantly reduces the volume of the search space and makes the brute force approach tractable. For the first frame, where the larval backbone is straight, we use the parameter vector obtained from the brute force approach as an initial guess and compute the parameter vector **p**_0_ that minimizes the cost function, using the *patternsearch* algorithm in MATLAB. For all the succeeding frames, the initial guess is the optimized parameter vector from the preceding frame.

In the succeeding iterations of coarse optimization, Lookup Table P is used to render the larval anterior and the larval posterior, given the parameter vector **p** (see **Materials and Methods: (b) Template-based pose estimation**: *Rendering larval projections*). During rendering, for pixels shared by the projection of overlapping larval anterior and posterior segments, the higher pixel intensity value is selected. The estimate **p**_0_ is computed by optimization of the cost function described previously. Since the Lookup Table P entries are rendered for discrete orientations of the larva, the estimate of **p**_0_ is coarse grained, and we find that the estimates of roll angle *γ*_0_ are often inaccurate. We thus pass **p**_0_ through a round of fine optimization in the local neighborhood of **p**_0_.

During fine optimization, we use **p**_0_ as the initial guess and search for local minima in a small neighborhood around this guess. Here, an image of the larval anterior is rendered by performing a refraction-based non-linear projection of the voxel-based model for every iteration (see **Materials and Methods: (b) Template-based pose estimation:** *Rendering larval projections*). Moreover, the optimization is performed for the anterior and the posterior independently to reduce the volume of the search space. In order to check for convergence, we then perform a final iteration of optimization over the 22-dimensional space and optimize the anterior and the posterior together. Since fine optimization is extremely slow (10–12 minutes /CPU/frame), we manually selected ~150 swim bouts each from free swimming, escape response and dark flash experiments for this task. The fits obtained from coarse optimization were visually inspected to select the 450 swim bouts.

#### (c) Neural network pose estimation

*Generation of ensemble of physical model poses*. A total of 35714 real larval poses were selected to generate the ensemble of physical model poses (**Fig 1C**), later used to generate the training dataset. These real larval poses are a subset of the poses estimated using the template-based approach (**Materials and Methods: (b) Template-based pose estimation**) that have a pose prediction accuracy greater than or equal to 0.9. We found that the set of larval poses was highly skewed, favoring poses with a nearly straight backbone (**Fig 1B** *Biased data*). Thus, before we could use these larval poses to generate the training dataset, we sought to resample the larval poses uniformly.

First, a kernel density estimate was performed using a Gaussian kernel to approximately infer the probability distribution of larval poses in the (<|Δ*θ*_i_|>, <|Δ*φ*_i_|>) space. In order to evaluate a reasonable bandwidth for the Gaussian kernel, we performed a search over 50 bandwidths uniformly selected in the range [0.01 rad, 0.1 rad]. The data were iteratively split into a test set and a training set in a 1:9 ratio. A kernel density estimate was evaluated using the training set and the iterable bandwidth. For each iteration, the log likelihood of the test set was calculated. The bandwidth that maximized the log likelihood of the test set was selected. The search for bandwidth was implemented using the *GridSearchCV* function of python’s scikit-learn (version 0.24.2) library. A 20-fold cross-validation was used to iteratively split the ensemble of real poses (**Fig 1A**) into test set and training set. The probability distribution ${℘}_{meanangle}(\langle \left|{\Delta \theta }_{i}\right|\rangle ,\langle \left|{\Delta \varphi }_{i}\right|\rangle )$ computed using the kernel density estimate was used to sample 2500 poses that are uniformly distributed in the (<|Δ*θ*_i_|>, <|Δ*φ*_i_|>) space (**Fig 1B** *Uniform data*). To achieve this uniform sampling, we choose 2500 poses, each with a probability inversely proportional to ${℘}_{meanangle}(\langle \left|{\Delta \theta }_{i}\right|\rangle ,\langle \left|{\Delta \varphi }_{i}\right|\rangle )$.

Next, we generated an ensemble of 500,000 physical model poses, which are modelled on real larval poses. Using the 2500 poses in the uniform data (**Fig 1B** *Uniform data*), we computed their probability distribution ℘_*angle*_ in the 18-dimensional space defined by Δ*θ*_1_… Δ*θ*_8_, Δ*φ*_1_… Δ*φ*_8_, *φ*_0_, γ_0_. The kernel bandwidth used to compute ℘_*angle*_ was inferred using the same procedure as described in the previous paragraph. We sampled 500,000 vectors conditional on ℘_*angle*_ and to each vector, assigned *x*_0_, *y*_0_, *z*_0_ and *θ*_0_ sampled from a uniform distribution. The range of (*x*_0_, *y*_0_, *z*_0_) was constrained by the dimensions of the imaging volume in the fish tank, while the range of *θ*_0_ was chosen as (−*π*, *π*]. The estimate of the kernel bandwidth obtained using our maximum likelihood approach described in the previous paragraph results in a continuous probability distribution that is a good approximation of the underlying data. This estimate serves as a guideline to the user for a suitable choice of the kernel bandwidth. However, a larger bandwidth can offer a more diverse dataset, possibly improving the network’s generalizability. (**S2 Table** shows the effect of a larger kernel bandwidth on the network’s pose prediction performance.)

*Preprocessing training dataset*. Using the ensemble of physical model poses, we generated a training dataset of 500,000 physical model images to train the neural network. Generation of all the physical model images was performed in 7 hours using MATLAB on 25 Xenon Gold 6150 CPUs. For every physical model pose, a set of three physical model images was rendered, as seen by the three cameras in our setup (**Fig 1D and 1E**), using the voxel based model (see **S1F Fig** and **Materials and Methods: (b) Template-based pose estimation**: *Physical model of the larva*) and the projection function (**S4 Fig**). The 20 fixed parameters of the physical model (see **Materials and Methods: (c) Neural network pose estimation:** *Physical model of the larva*) were estimated using template-based optimization. While rendering physical model images, the intrinsic parameters of the voxel-based model were varied uniformly randomly by ±5% around their estimated values. All the physical model images were then pre-processed before passing to the neural network.

More specifically, the three 2-D larval projections corresponding to each of the 500,000 physical model poses were rendered onto three 141x141 images with pixel values scaled between 0 and 1. The larval projections were displaced at random by 0–20 pixels, both horizontally and vertically. To each image, we added Gaussian noise, whose mean and standard deviation were sampled randomly in order to mimic the background noise in the real images. The brightness at each background pixel of the projections of the bottom camera and side cameras is sampled from Gaussian distributions *N*(*m*_*bottom*_, *s*_*bottom*_) and *N*(*m*_*side*_, *s*_*side*_), respectively. For each projection of the bottom camera, the mean *m*_*bottom*_ of this Gaussian distribution is a product of two random variables—*X*_*μ*,*bottom*_, drawn from a uniform distribution between 0 and 1/255 and *Y*_*μ*,*bottom*_, drawn from a Gaussian distribution with mean 50 and standard deviation 10, while the variance, *σ*^2^_*bottom*_ of the Gaussian distribution is a uniform random variable between 20/(255)^2^ and 70/(255)^2^. For each projection of the side camera, the mean *m*_*side*_ of the Gaussian distribution is a product of two random variables—*X*_*μ*,*side*_, drawn from a uniform distribution between 0 and 1/255 and *Y*_*μ*,*side*_, drawn from a Gaussian distribution with mean 20 and standard deviation 10, while the variance, *σ*^2^_*side*_ of the Gaussian distribution is a uniform random variable between 10/(255)^2^ and 60/(255)^2^. Next, we rescaled the pixel values between 0 and 255, to mimic the format of the recorded images–unsigned 8-bit.

The sampling of physical model poses in the ensemble of physical model poses, varying of the 20 fixed parameters of the physical model and the downstream preprocessing of the physical model images makes our training dataset diverse, generalizing it beyond the features of our imaging data. It is better to have an overly diverse training set, even if not all its poses are weighted heavily in neural network outputs, than to have an under-representative training set.

*Preprocessing real data*. The real images were preprocessed before passing as inputs to the neural network. We performed background subtraction and cropping on every frame (see **S7 Fig** and **Materials and Methods: (b) Template-based pose estimation:** *Preprocessing*) obtained from the recorded videos to validate the trained neural network. The neural network accepts three images each of dimensions 141×141. Since the cropped 2-D larval images were of varying size, they were also padded appropriately with zeros to dimensions 141×141. In order to simulate background noise in the padded region, we first generated a background vector of pixel values along the pre-processed image’s border. This border was defined by the region with 5 pixels of the image boundary. The padded region was assigned pixel values from this vector, selected uniformly at random. This procedure of simulating the background noise works in most cases because the background distribution is uniform, i.e. the distribution of the intensity in the border is the same as that in any other region of the image not occupied by the larva. In some frames, there is a possibility of an irrelevant larva (from the context of pose estimation) occurring in the 5-pixel border. In order to avoid including pixels corresponding to a larva in the background vector, we excluded all pixels that have a value greater than 3*σ* with respect to the background vector’s mean. Notably, this procedure is not ensured a 100% success in simulating the background. We find rare cases where the padded region’s noise distribution does not represent the background noise distribution of the pre-processed image. The network predictions are inaccurate for such frames. One could overcome this issue by originally cropping 141×141 windows around the larva before performing pre-processing. However, such cropping dimensions may not be practical to achieve in cases where the larva occupies a region near the frame’s border. For our network evaluation, we challenge the network model by performing the above padding procedure for all frames, essentially assuming the worst-case scenario of the larva always being in the border region.

The comparison of template-based pose estimations and neural network pose estimations (see **S8 Fig**) was performed over 424 swim bouts (41756 frames), for which the computationally expensive template-based pose estimates were evaluated. For a fair comparison between the template-based pose estimations and the neural network pose estimations (see **S8 Fig**), we manually discarded all frames that had more than one larva in the cropped preprocessed images (see **Materials and Methods: (b) Template-based pose estimation:** *Preprocessing*) passed as input to the convolutional neural network. Since the network model is only trained with a training dataset involving single larvae, we observed that the network predictions are inaccurate when there are multiple larvae in the cropped preprocessed input image.

While analyzing our entire dataset of 630 movies (68181 frames) to assess the network’s performance (see **Fig 2**) and larval swimming kinematics (see **Figs 4 and 5**), the issue of having multiple larvae in the cropped preprocessed input image was resolved by first using a binary mask of the larva of interest and assigning all the background pixels an intensity of 0. Realistic background noise was then artificially rendered by the procedure described above. The larval mask was obtained by using the first round of optimization of the template-based pose estimation (see **Materials and Methods: (b) Template-based pose estimation**: *Optimization*), and rendering a physical model image based on the estimated pose. Pose estimation using the first round of optimization takes 2 minutes per frame with reasonably accurate pose estimates. This means that some of the inaccurate larval masks used to remove any irrelevant larvae also remove parts of the larva of interest. Neural network pose predictions corresponding to such cases are inaccurate and are automatically filtered out by setting a threshold on the accepted prediction score for analysis. We choose a threshold of 0.85 for our analysis in **Figs 4 and 5**.

Here we emphasize that we do not expect the user to manually discard frames having multiple larvae in the cropped preprocessed input image to generate pose estimations. We also do not propose generation of larval masks to remove irrelevant larvae from the cropped preprocessed input image. Both these procedures are performed in these cases for a fair assessment of the network’s performance. In order to estimate the larval pose directly from video recordings and only using the neural network, a user may use one or both of the two approaches below:

1. perform experiments with low larval density, such that individual larvae can be cropped easily;
2. automatically discard network outputs for cropped preprocessed input image containing multiple larvae, based on a cut-off on the pose prediction score.

*Convolutional neural network model*. Our network consists of an encoder of four bottleneck residual blocks as implemented in Ref. [35] with 32, 64, 128, and 256 channels, respectively. The convolutional operations involved kernel size = 3×3, stride = 1 and padding = 3. The encoder is followed by a decoder, consisting of three fully connected layers of dimensions 1×288, 1×144, and 1×72, respectively. The output of the decoder is passed through a sigmoid layer and scaled between 0 and 141. This scaled output (a 1×72 vector) is reshaped into three 2×12 matrices, corresponding to the 2-D projection of the larval pose on the three camera views. Every convolutional and fully connected layer is preceded by a batch normalization [70] layer and succeeded by a leaky rectified linear unit activation function [71] (negative slope = 0.01). The network was trained for 120 epochs using the Adam optimizer with a learning rate of 0.001 and a batch size of 300. The convolutional neural network was implemented using the PyTorch library (version 1.10.1) and trained in parallel on 2 NVIDIA V100 GPUs [72]. The network is completely trained in 5–6 hours. Network validation was performed on a single NVIDIA A100 GPU.

The loss function for training the network consists of two terms: ‘backbone loss’ and ‘eye loss’. The backbone loss is simply the root mean squared error between the 10 2-D projection pose coordinate predictions for the three camera views (3×2×10 vector) and the corresponding ground truth. The eye loss is similarly defined by the root mean squared error between the 2 2-D eye coordinate predictions for the three camera views (3×2×2 vector) and the ground truth 2-D eye coordinates, with a small modification: the eye loss is made symmetric with respect to the eye indices. More specifically, the eye loss is defined as:

$$\sum_{\alpha =1}^{3}min(L_{\alpha }({eye}_{1p},{eye}_{1g}),L_{\alpha }({eye}_{1p},{eye}_{2g}))$$

where subscripts 1 and 2 for the eye correspond to the 11^th^ and the 12^th^ coordinate of the 2x12 dimensional 2-D projection pose coordinates, subscripts *p* and *g* for the eye correspond to the network prediction and the ground truth and the ℒ_*α*_ corresponds to the root mean squared error for camera *α*. The total loss function is defined as (backbone loss) + λ*(eye loss), where *λ* captures the relative importance of the eye loss with respect to the backbone loss. For all our neural network experiments, we set *λ* = 5.

*Triangulation of 3-D pose coordinates*. The ten 3-D backbone coordinates are triangulated using the projection function and the 2-D projection pose coordinates obtained as the neural network’s output. Each of the ten points is triangulated independently by minimizing the least square error between the 2-D coordinate obtained from the neural network and the 2-D projection of the triangulated coordinate obtained using the projection function. We use the center of the tank as the initial guess for this coordinate and implement the optimization using the *least_squares* function of the *scipy*.*optimize* package (version 1.7.3) in Python 3.9.9.

The coordinates of the eyes cannot be triangulated directly because of the symmetry in the loss function with respect to the eye coordinates. The symmetry in the loss function causes an ambiguity in the indices of the eyes in the network’s predicted 2-D projection pose coordinates. For example, the network may predict the right-eye as the 11^th^ 2-D coordinate in its 2x12 output vector for the bottom camera, but the 11^th^ coordinate in the output vector for one of the side cameras may be the left-eye. In such a case, one cannot directly triangulate the 11^th^ coordinates in the three views to obtain the 3-D coordinate of one of the eyes. This ambiguity of eye indices is tackled by first permuting over all 8 combinations of eye indices and triangulating the 3-D coordinates of the two eyes in each case. The indices that result in the least triangulation error are accepted. This ambiguity of eye indices is tackled by first permuting over all 8 combinations of eye indices and triangulating the 3-D coordinates of the two eyes in each case. The indices that result in the least triangulation error are accepted.

The ambiguity in the indices of the eyes caused by the symmetry in the loss function with respect to the eye coordinates also means that there is no notion of left and right eye. This makes the assignment of roll angle *γ*_0_ impossible. To assign the roll angle *γ*_0_ in all frames, we assume that the larva never swims belly up. This reintroduces asymmetry in the two eyes, allowing for a unique *γ*_0_ to be assigned for the given prediction of the eye centroid. The assumption could be relaxed to allow belly-up orientation if one examines belly vs. eye height.

#### (d) Modeling external datasets

To test the robustness of our method, prediction was performed on 2-D datasets and 2-camera 3-D datasets that were collected by us and obtained from other groups [37–39]. Our 2-camera 3-D dataset was created by simply using our original 3-D dataset and discaring all images obtained from camera 2.

The workflow for pose estimation of the 2-D and 2-camera 3-D datasets was essentially the same as that used in 3-D pose estimation, with slight differences in the training dataset generation and the convolutional neural network model architecture (see **S1 Text. Supporting Methods: *Modeling external datasets*** for a detailed description of these workflows). Briefly, for 2-D pose estimation, the training dataset was generated using a new ensemble of physical model poses that was inferred using template-based pose estimation on our 2-D dataset (please refer **S2D Fig**). For 2-camera 3-D pose estimation, we used the same ensemble of physical model poses used for 3-D pose estimation (**Fig 1**). For each physical model pose, a single physical model image was rendered for the bottom camera. For the 2-camera 3-D external dataset [37], two images were rendered using the appropriate mapping from the lab frame to the camera frame for each of the two cameras (see **S2C-S2F Fig**, [37] and **S1 Text. Supporting Methods: *Modeling external datasets***). Three training datasets were generated for pose estimation on (1) all 2-D datasets, (2) 2-camera 3-D external dataset, (3) our 2-camera 3-D dataset. A convolutional neural network model was trained for each of the datasets. The model’s architecture was similar to the one used for our 3-D pose estimation workflow (see **Neural network pose estimation:** *Convolutional neural network model*), with small differences to account for the change in the size of the network’s input and output vectors. The size of the fully connected layers in the decoder module were modified to obtain the required vector outputs representing 2-D pose projection coordinates (see **S1 Text. Supporting Methods: *Modeling external datasets***). All network models were trained for 100 epochs.

The input images were preprocessed (see **S2B and S7 Figs**) and passed through the trained convolutional neural network (see **S2H Fig**) to obtain 2-D pose projection coordinates. For 2-camera 3-D pose prediction, the 3-D pose was reconstructed using the mapping from camera frame to lab frame (see **S1 Text. *Modeling external datasets*** and **Materials and Methods(c) Neural network pose estimation:** *Triangulation of 3-D coordinates*). The predicted poses were evaluated using Pearson’s correlation coefficient as described previously in our 3-D pose estimation workflow (see **Fig 2C** and **Results: Convolutional neural network model trained on physical model images performs fast and accurate pose estimation on real images**: *Model evaluation*).

#### (e) Statistical analysis

*Statistical significance of the observed difference between two distributions*. We performed statistical tests to check the significance of the differences between two given distributions (see **Convolutional neural network model trained on physical model images performs fast and accurate pose estimation on real images**: ***Model evaluation***, **Figs 4, 5 and S8,** and **3-D kinematic parameters reveal unique features of larval swims across the three experiments**). To calculate the statistical significance that the mean of one distribution (with *n* samples) is greater than that of the other (also with *n* samples), we posed the null hypothesis that the samples belonged to the same underlying distribution. The p-value of the test was calculated as the probability of obtaining the observed or a larger difference of means *μ*_1_ and *μ*_2_, given the above null hypothesis. (Here, *μ*_1_>*μ*_2_, without loss of generality.) Combining the two samples into a single distribution, we used bootstrapping to generate a distribution of means using *P*. We sampled two distributions of size *n* uniformly at random and calculate the difference of their means. We repeated this computation of difference of means of two sampled distributions *N* = 10000 times, resulting in a distribution of difference of means, *D*_*μ*_. The p-value of the test was computed as the cumulative probability *p* = Pr(*D*_*μ*_≥*μ*_1_−*μ*_2_). We used the same approach to test the statistical significance of differences in the lower and upper quartiles of all distributions in **Figs 4** and **5**.

*Statistical significance of asymmetry*. To test the statistical significance of asymmetry in a given distribution between its positive and negative samples (distributions in **3-D kinematic parameters reveal unique features of larval swims across the three experiments** and distributions in **Fig 4**), we calculated the probability of obtaining the observed fraction of positive and negative samples or a higher fraction of the same, under the null hypothesis that the data comes from a binomial distribution with *p* = 0.5. A similar test was used to test the predominance of dataponts in the second quadrant in **Fig 5D** with *p* = 0.25.

## Supporting information

S1 Text Supporting Methods. (DOCX)

S1 Table Camera calibration parameters: Intrinsic and extrinsic matrices illustrating the camera parameters used for analysis. The extrinsic matrices **M** shown below encode the orientation and location of the cameras, where the translation vector T is estimated in mm. The intrinsic matrices **K** encode the intrinsic parameters of the camera in pixels (1 pixel = 7.4 μm). The two matrices were used to analyze fish swimming experiments and had to be calibrated periodically to account for drift in the cameras.
(DOCX)

S2 Table Comparison of convolutional neural network model pose predictions on different datasets: Convolutional neural network models trained on different datasets and average pose prediction scores on real data from the three experiments combined. (DOCX)

S3 Table p-values from comparing the ranges of different kinematic parameters between different experiments. The upper (lower) left triangle of each sub-table compares the upper (lower) quartiles of each parameter across the three experiments: free swimming, acoustic startle, and dark flash.
(DOCX)

S1 Fig **Template-based algorithm tracks 3-D zebrafish swimming poses** (a) Movies of larval swims are acquired at 500 fps by three cameras oriented perpendicular to each other. (b) For each frame, the larval position is triangulated, and the three camera projections are cropped. (c) A model for the larval backbone is generated using 9 rigid segments of equal length oriented at different angles based on an initial guess **p**_**0**_. Two frames of reference define **p**_**0**_: (*x*_lab_, *y*_lab_, *z*_lab_) defines the lab reference frame with its origin fixed to the tank’s center and the three axes defined by the principal axes of the cameras. The Euler angles of the larval anterior (*θ*_0_, *φ*_0_, *γ*_0_) are defined with respect to this fixed lab reference frame. (*X*, *Y*, *Z*) defines the fish frame of reference fixed to the larval head, with the *X*-axis perpendicular to the line joining the center of the eyes and the *XY*-plane defined by the plane of the eyes. The shape of the larval backbone is parameterized in this reference frame by Δ*θ*_*i*_ and Δ*φ*_*i*_ (see Δ*θ*_7_ and Δ*φ*_7_ for example). Optimization of the parameter vector **p** occurs in two rounds: *Coarse Optimization* and *Fine Optimization*. (d) Coarse Optimization: The coordinates of the backbone are projected on the three camera planes using their respective calibrated parameters, taking into account refraction. (e) The projections in (d) are used to compute the indices of the appropriate ‘Lookup Table P’ entry rendering a digital projection of each segment of the larva. (f) Fine Optimization: A voxel-based 3-D model is rendered on the three camera planes using the camera calibrated parameters. This step allows a parameter search over **p** at a higher resolution than that achieved using Lookup Table P. (g) The three digital projections are positioned on images with the same resolution as their respective real projections in (b). (h) The sum of the squared pixel values of the three difference images is used as the cost function during optimization. (i-h) The optimization is run until convergence for each frame in parallel to obtain an ensemble of larval poses.
(PNG)

S2 Fig Pose estimation workflow. (a) Video recordings of larval behavior are obtained using a 2-D or 3-D imaging system. (b) Larval images are preprocessed: *Background subtraction*: The raw image is subtracted from the background image. *Median filtering*: A median filter of kernel size [5,5] is convolved over the image to reduce noise. *Cropping*: A bounding box is cropped around the larva. *Resizing*: The cropped image is resized so that the length of the larva is within the range of lengths used to generate the physical model images in the training dataset. (c) A projection function mapping the 3-D lab frame coordinates to 2-D pixel coordinates is empirically determined. (d) An ensemble of physical models is sampled using the ensemble of real larval poses. We have generated a representative ensemble of real poses for 2-D and 3-D larval motion. The appropriate ensemble must be selected based on the imaging setup (2-D or 3-D) used in (a). (e) The fixed parameters of the physical model are estimated. We have shown that this step is not a strict requirement as training datasets generated with a certain set of fixed parameters generalize for pose prediction on datasets in different imaging conditions. (f) A training dataset of physical model images and their underlying pose annotations is generated. (g) A convolutional neural network model is trained to learn the relationship between physical model images and the 2-D projection pose coordinates. (h) Pose prediction is performed by passing the preprocessed images (b) as input to the convolutional neural network model. The larval coordinates in the lab reference frame are evaluated using the 2-D projection pose coordinates using a mapping from camera frame to lab frame. (i) Pose evaluation is performed by comparing the physical model images resulting from the neural network’s predicted 3-D pose and the preprocessed input in (b) passed as input to the trained network model. The comparison is quantitatively computed as Pearson’s correlation coefficient.
(PNG)

S3 Fig Experimental setup for high-throughput video recording of 3-D larval behavioral experiments. (a) Experimental setup for 3-D experiments. Zebrafish larvae swimming freely in a 7 x 7 x 7 cm glass tank are imaged using three cameras at 500 fps under infrared illumination. (b) Schematic of the setup used to provide acoustic stimulus. Larvae are habituated to white light using a white LED for at least 4 minutes. A dark flash stimulus is provided when the three cameras simultaneously detect a unique larva in their field of view. The instantaneous deactivation of the white LED marks the onset of the dark flash stimulus. (c) Schematic of the setup used to provide acoustic stimulus. The stimulus is generated by dropping a cylindrical weight on the platform supporting the tank from a suitable height. The acoustic signal propagating through the cube startles the larva. The time of the stimulus is recorded using an infrared laser projected in the field of view of one of the cameras. The weight is pulled back up using an Arduino-controlled servo motor and held in place using an electromagnet until the next round of stimulus.
(PNG)

S4 Fig 3-D triangulation from 3 camera views with refraction calibration. (a) Images of the calibration dot pattern in air. (b) Images of the same calibration pattern inside the glass cube filled with water. The position of the pattern is identical in both sets of images. (c) A sample image showing the coordinates of the dots on the images taken by one of the cameras. Green dots correspond to an image in (a) (camera 1), in the absence of refraction. Red dots correspond to an image in (b) (camera 1), in the presence of refraction from the glass cube and water. (d) The projection function (*f*_1*x*_(*y*_*lab*_, *z*_*lab*_)) for the coordinates *x*_1_ in camera 1 of the dot pattern, and fit to a cubic function. The surface plot color represents *x*_1_ for visual convenience. (e) Triangulation error of the lab coordinates of the dots on the calibration pattern from the images taken in water. The mean error is 1.07 mm without refraction correction and 0.03 mm with correction.
(PNG)

S5 Fig Non-linear projection parameters used to generate Lookup table R. (a) Ray CI emanates from the centroid of the larva, C, and passes through an arbitrary point I in the tank. By invoking ray optics, for every pixel S of the camera sensor array, there is a unique ray CI emanating from a point C inside the tank that is incident on S after refraction at the water-tank-air interface. (b) Lookup Table R stores the two vectors **a** and **r** that uniquely define ray CI for every pixel of the camera sensor, for all three cameras.
(PNG)

S6 Fig Generation of Lookup Table P entries storing 2-D views. (a) Rendering the larval anterior. Lookup Table P entries of the anterior are generated using orthographic projections of a voxel-based model along the principal axis of each camera. Given the parameter vector **p**, the indices of the appropriate lookup table entry are determined to render the digital projection. The 2-D projection of the larval backbone (green chain) is computed using the nonlinear projection parameters (top panel). Ray CI emanates from the 3-D larva’s centroid and is incident on its 2-D projection. The appropriate lookup table entry to be used is determined by rotating CI such that it is parallel to the principal axis of the corresponding camera (bottom panel). (b) Rendering a tail segment. Image 1 shows a digitally rendered component of a tail segment. Images 2 and 3 show entries in the lookup table adjacent to the entry for image 1. The tail segment in image 2 is 0.2 pixels lower than that in image 1. The segment in image 3 is that in image 1 rotated by 5° clockwise. (c) Rendering the larva. Scheme for the construction of a grayscale fish model from the lookup table. Only a small fraction of the tables is shown.
(PNG)

S7 Fig Pre-processing of raw images for pose estimation using physical model-trained convolutional neural network model. (a) Example of grayscale 8-bit images captured by the 3 orthogonal cameras for a free swimming experiment. (b) Each of the three camera images are background subtracted independently. A background image is created by computing the 90th percentile intensity of every pixel over the entire video. The raw image is subtracted from the background. (c) Background subtracted images for each of the three camera views are independently segmented. The background subtracted object is passed through a Gaussian filter (window size: 5x5 pixels, standard deviation: σ = 1) to reduce high frequency noise. Larvae are detected in each camera image independently using a binary threshold obtained using Otsu’s algorithm and scaling it such that larvae can be consistently detected across all frames. Segmented blobs corresponding to larvae detected simultaneously in all the three camera views are detected. The detection is performed using all the centroids (*x*_0*i*_, *y*_0*i*_) of the blobs (centroids indicated by red dots) detected in camera *i* (*i* = {1,2,3}) and the 2-D to 3-D mapping determined during camera calibration. (d) Cropped preprocessed images of a unique larvae (length = 3.8 mm) seen by the three cameras. The cropped images are passed as input to the convolutional neural network model.
(PNG)

S8 Fig Network predictions are invariant to the amount of training data and comparable to template-based estimates The performance of the CNN method is not affected by using a smaller subset of real poses to generate the ensemble of physical model poses. The distributions of predictions scores are visualized using kernel density estimate over pose prediction scores obtained using different approaches across free swimming, acoustic startle and dark flash experiments. The distributions compare pose prediction accuracy of network models trained using different subsets of the ensemble of real poses and that of the physical model fits. The mean of each distribution is shown using overlaid dashed lines. Using only 25% (*N* = 8929) of poses from the ensemble of real poses to generate the training dataset does not deteriorate network predictions, compared to the performance of a network trained using 100% (*N* = 35714) of poses from the ensemble of real poses (compare pink and green distributions and their means–overlaid dashed lines). When the training dataset is generated using only free swimming poses from the ensemble of real poses (*N* = 9536), the network performance for acoustic startle and dark flash experiments declines marginally (purple distributions and overlaid dashed lines). The template-based pose estimates are marginally better than neural network predictions in the acoustic startle experiments and consistently worse in dark flash experiments. On average, the network trained using 100% of the data (mean prediction score = 0.91) is comparable to the template-based predictions (mean predictions score = 0.90).
(PNG)

S9 Fig Validation of our pose estimation approach on our data in different experimental contexts. (a) Example sequences of raw images from our datasets, and superimposed 2-D projected poses from our neural network: ten 2-D backbone coordinates (green) and two 2-D centroids of eyes (red). (i) Free swimming bout in 2-D (larval length = 3.9 mm). (ii) Acoustic startle swim bout in 3-D, showing only the camera 1 and 2 views (larval length = 3.9 mm). The convolutional neural network model was trained on camera 1 and 2 only (see **S1A Fig**). (b) A histogram of pose prediction scores for all frames of the swim bouts are to the right of each example.
(PNG)

S10 Fig Larval swimming dynamics comprise 3-D bending modes. Singular Value Decomposition of **ϴ**(*t*) from all swim bouts identifies orthogonal bending modes or “eigenshapes” of the larvae (see Ref. 23). Eigenshapes are arranged in decreasing order of their contribution to the total variance in **ϴ**(*t*). Eigenshapes 1, 2, and 4 (Lateral Eigenshapes—black) mainly contribute to lateral bending motion (Δ*φ*_*i*_ ~ 0), while eigenshapes 3 and 5 (Dorsal Eigenshapes—gray) mainly contribute to dorso-ventral bending motion (Δ*θ*_*i*_ ~ 0). The blue curve indicates the cumulative contribution of the first several eigenshapes to the total variance in **ϴ**(*t*).
(PNG)

S11 Fig Realistic projections of multiple larval zebrafish are rendered for every camera. Physical model projections of multiple larvae are rendered as an extension of our approach. A large dataset of such images can be potentially used to develop pose prediction workflows to study social behavior of larval zebrafish. The network architecture and loss function used for such a workflow when fish number varies from frame to frame may be adapted from successful models developed for multi-animal pose estimation task (59–61).
(PNG)

S1 Video An example of 3-D reconstructed larval swim bout recorded in the acoustic startle experiment. (MP4)

S2 Video Examples of physical model poses sampled from the ensemble of physical model poses. (MP4)
