# Supplementary material for: Rapid automated 3-D pose estimation of larval zebrafish using a physical model-trained neural network
Source: PLoS Comput Biol. 2023 Oct 23;19(10):e1011566. doi: 10.1371/journal.pcbi.1011566 (PMC10621986; doi:10.1371/journal.pcbi.1011566)
Supplement: S1 Text — (DOCX) [file pcbi.1011566.s001.docx]

“Rapid automated 3-D pose estimation of larval zebrafish using a physical model-trained neural network”

Aniket Ravan^1,2^, Ruopei Feng^2,3^, Martin Gruebele^1,2,3,*^, Yann R. Chemla^1,2,4,*^

**Supporting Methods**

***Lookup Table P***

Lookup Table P of larval projections is used to efficiently render larval projections. Instead of rendering a 3-D digital larva in every iteration of the optimization, we store an array of possible larval projections in a lookup table that can be accessed quickly at every iteration. This lookup table broadly consists of two parts: projections of larval anterior (eyes, head and belly) and projections of larval posterior (tail segments). During the first round of optimization (see **Materials and Methods: (b) Template-based pose** estimation: *Optimization*), we use both parts of the lookup table (projections of anterior and posterior) to completely render the larva. During the second round of optimization (see **Materials and Methods: (b) Template-based pose estimation**: *Optimization*) and while generating physical model images used to train the neural network model (see **Figure 1e**), only the projections of larval posterior are rendered using Lookup Table P, while the larval anterior is rendered directly from the voxel-based model (see **Materials and Methods: (b) Template-based pose estimation**: *Physical model of the larva*).

We note that the 2-D larval projections are a function of both the orientation and position of the larva in the tank. A brute-force construction of the lookup table indexed by the position and orientation of the larva in the tank would require too much storage memory. (For example, if the lookup table stores entries corresponding to the larva located in *ℓ* positions along each direction, the dimensions of the lookup table scales as *ℓ^3^.* Given that a displacement of 0.3 mm in the tank causes a visually recognizable change in the projection and that the field of view is 3 x 3 x 3 cm, *ℓ* has to be at least 10.) Such a lookup table would also generate many degenerate projections. As detailed below, we instead generate a ‘Lookup Table P’ that is indexed by as few variables as possible, and whose size is minimized by exploiting rotational and inversion symmetries.

As with the physical model of the larva, the anterior of the fish (head, gut) and posterior (tail) each have their own Lookup Table P and indexing schemes. For the anterior, each lookup table entry is generated from orthographic projections of the 3-D voxel-based physical model (see **Materials and Methods: (b)** **Template-based pose estimation***: Physical model of the larva*; **S1 Figure** and **S5a Figure**). It is indexed by only 6 variables: the orientation of the larva (*θ*_0_, *φ*_0_, *γ*_0_), a scaling parameter *l*_A_, and sub-pixel offsets (*δ_x_*, *δ_y_*) of the projection. *l*_A_ accounts for the size of the projection, which varies with the position of the larva in the tank. The array of lookup table entries for the bottom and two side cameras are constructed independently to account for the differences in illumination and sizes of larval projections resulting from the bottom camera being closer to the tank compared to the side cameras. Each entry of the lookup table for the bottom camera has a resolution of 49 x 49 pixels and the table has 18 entries of *l*_A_, 180 entries of *θ*_0_ in the range [0, π] (entries for the remaining two quadrants can be generated using rotation and/or inversion), 11 entries of *φ*_0_ in the range [0, π/2] (entries for the remaining three quadrants can be generated using rotation and/or inversion and correction for subpixel offset), 11 entries of *γ*_0_ in the range [-5π/12, 5π/12], and 5 entries each for subpixel offsets of 0.2 pixel steps in two orthogonal directions. The lookup table for the side cameras has 18 entries of *l*_A_*,* 21 entries of *θ*_0_ in the range [0, π], 121 entries of *φ*_0_ in the range [-π/2, π/2] (entries in the remaining quadrants can be generated by rotation and/or inversion), and 5 entries each for subpixel offsets. The step size of the angles is decided such that the length of the 2-D projection of the anterior increases by a constant amount as one iterates over the angles. This ensures that the space of 2-D projections of larval heads is homogeneously sampled. Integral values of pixel offsets are degenerate.

As the 2-D projections of the larval posterior are not rendered using a voxel-based model, Lookup Table P is indexed differently for the posterior than for the anterior. The lookup table stores 2-D projections of tail segments indexed by 5 variables: the segment number, length of the 2-D segment *l_T_* (7 entries), subpixel offsets of 0.2 pixel along the horizontal and vertical direction in the image (5 entries each), and the orientation of the 2-D segment in the plane (360 entries). The tail segment in the physical model does not have a roll angle because the larval tail is approximately cylindrically symmetric. The subtle changes in the tail’s projection that might occur due to torsion are not discernable in our images, constraining the tail segments in our physical model to be torsionally rigid. By construction, the tail segments in Lookup Table P can be accessed directly using the 2-D projection of the larval backbone (see **Materials and Methods: (b)** **Template-based pose estimation:** *Rendering larval projections* below).

***Rendering larval anterior using Lookup Table P***

To render the larval anterior from Lookup Table P, we first determine the indices corresponding to the orientation. We note that the angles (*θ*_0_, *φ*_0_, *γ*_0_) corresponding to the indices used during construction of Lookup Table P are not the same as the angles (represented in **p**) defining the orientation of the physical model of the larva, if the 2-D projection of the real larva and the orthographic projection of the voxel-based larva are to match (with the exception of the rare case where the larva is in the center of the tank). This difference occurs because Lookup Table P, in order to increase computational speed (see **S1 Text: Supporting methods*: Lookup Table P***), is constructed using orthographic projections of the voxel-based model. Thus, as an example, for a larva oriented horizontally facing toward the camera and positioned at a certain height above the principal axis, it should be possible to see the projection of the larval belly and head on the 2-D image projected on the camera. In order for the voxel-based model to produce the same 2-D image rendered by orthographic projection, a small positive pitch angle needs to be introduced to the model. As a result, the indices corresponding to the orientation (*θ*_0_, *φ*_0_, *γ*_0_) need to be adjusted based on the position of the larva in the tank.

To determine the adjusted orientation indices, we assume that all the rays emanating from each voxel of the larval anterior that are incident on their respective pixel in the 2-D projection are parallel. We make this assumption since the size of the larva is negligible compared to its distance from any of the cameras. We further assume that the direction of all these parallel rays is the same as that of the ray CI emanating from the anterior’s center of mass (**S5a Figure**). Under these assumptions, the adjusted orientation is determined from rotating the larva such that CI is parallel to the principal axis of the camera of interest. To carry out this rotation, we find an arbitrary point I on ray CI, defined as the intersection of ray CI and the plane perpendicular to the principal axis of the camera and passing through the center of the tank. We then find the elevation angle and the azimuthal angle of CI. We note that a set of five points representing the centroid of the larval eyes, belly, head and the larval centroid uniquely define (*x*_0,_ *y*_0,_ *z*_0,_ *θ*_0_, *φ*_0_, *γ*_0_). These five points are rotated through two Euler rotations such that the azimuthal angle and elevation of CI is zero. This changes the parameters (*x*_0,_ *y*_0,_ *z*_0,_ *θ*_0_, *φ*_0_, *γ*_0_) that the transformed five points represent. An orthographic projection of the voxel-based model that has the same orientation as this new set of (*θ*_0_, *φ*_0_, *γ*_0_) will project and occlude similar parts of the larval anterior as those in the real projection. The appropriate lookup table entry corresponding to the yaw, pitch, and roll (*θ*_0_, *φ*_0_, *γ*_0_) of the reoriented model is then used to represent the projection of the larval anterior. The scaling parameter *l_A_* can be determined using trigonometry, given the 2-D projection (and thus, the length) of the head segment and the orientation of the voxel-based model whose orthographic projection is to be invoked.

***Modeling external datasets***

*Model training*

Performing pose estimation on 2-D datasets required generating a 2-D dataset of physical model images and training a physical model-trained convolutional neural network model. The workflow of generating the training dataset was similar to that used for generating the 3-D training dataset (see **Figure 1a-h**) and thus required estimating an ensemble of real poses obtained from 2-D experiments (see **S2d Figure**). We performed pose estimation using the first round of template-based pose estimation (see **Materials and Methods: (b) Template-based pose estimation:** *Optimization*). Larval projections for this optimization were generated using a Lookup table analogous to Lookup Table P (see **Materials and Methods: (b)** **Template-based pose estimation:** *Lookup Table P*), generated by setting $\varphi_{0}, \gamma_{0}, \varphi_{i}$ and $z_{0}$ to zero. We obtained 184473 2-D larval poses, forming the ensemble real poses for 2-D larval motion. Each pose was completely defined by 12 parameters ($x_{0},y_{0},\theta_{0},\theta_{i}),$where $i\in\left[ 1,8 \right].$ We sampled 5000 poses uniformly at random, which were further sample (see our approach in the 3-D workflow in **Figure 1a-c** and **Results**: **Convolutional neural network model trained on physical model images performs fast and accurate pose estimation on real images**: *Generation of training dataset*) an ensemble of physical model poses that uniformly represented 500,000 poses with different amounts of mean 2-D backbone curvature ($<|\theta_{i}|>$). For computational efficiency, physical model images were rendered using orthographic projections of the voxel-based physical model (see **Materials and Methods: (b) Template-based pose estimation***: Rendering larval projections*, *Physical model of the larva*). The fixed parameters of the physical model (see **Materials and Methods**: **(b) Template-based pose estimation**: *Physical model of the larva*) were determined using an optimization using the *patternsearch* algorithm in MATLAB (see **S2e Figure**). This optimization was performed by minimizing the sum of squared differences between the rendered physical model image and a manually picked image of a straight larval backbone. Manually picking larval images with a straight backbone for this task ensured that the adjustable parameters (see **Materials and Methods**: **(b) Template-based pose estimation**: *Physical model of the larva*) $\theta_{i}$ of the physical model (in addition to $\varphi_{0}, \gamma_{0}, \varphi_{i}$ and $z_{0}$) could be fixed to zero. While rendering the images (see **S2f Figure**), the fixed parameters were varied uniformly at random around the results obtained through *patternsearch* to make the physical model images diverse. Gaussian noise was added to the rendered 2-D physical model images and a bounding box around the larva. The distance of the edge of the bounding box from the boundary of the fish was randomly varied between 0 and 30 pixels, to account for variations in the sizes of cropped raw images occurring while performing pose predictions (see paragraph below). The length of the fish was varied between 65 pixels and 85 pixels uniformly at random, to reflect on the observed variation of fish lengths in our 2-D imaging data. The training and validation dataset was obtained by splitting a set of annotated 500,000 rendered physical model images into a ratio of 9:1 respectively.

We trained a convolutional neural network model using this dataset (see **S2g Figure**) and performed pose projections on our 2-D larval recordings and two external datasets of larval locomotion in a 2-D arena. The convolutional neural network used for 2-D pose predictions accepts a preprocessed cropped image of the larva and outputs 12 2-D projection pose coordinates (see **Results**: **Convolutional neural network model trained on physical model images performs fast and accurate pose estimation on real images: *The 3-D physical model of the larva***).

Similar to the convolutional neural network model used for 3-D pose predictions, the network model used for 2-D pose prediction consists of two modules: an encoder and a decoder. The encoder consists of four bottleneck residual blocks as implemented in (1) with 32, 64, 128, and 256 channels respectively. The decoder consists of three fully connected layers of dimensions 1x192, 1x96 and 1x24. We used a leaky rectified linear unit (2) as the non-linear activation function after every layer. The non-linear activations were preceded by a batch normalization layer (3). The output is reshaped into a 2x12 array of 2-D projection pose coordinates, which are the pixel coordinates of 10 equally spaced points along the larval backbone and 2 points representing the centroid of the eyes. The convolutional neural network model was trained for 100 epochs.

*2-D pose estimation: Pose prediction*

Pose predictions were performed on all external 2-D datasets using the parameters of this single trained convolutional neural network model (see **S2h Figure** and **S7 Figure**). For performing network predictions, the input images for the network were obtained by preprocessing raw images (see **S2a,b Figure**) background subtracting, median filtering (kernel size: [5,5]) and cropping a bounding box around the larva with a minimum space of 15 pixels between the larva and each side of the bounding box. The resulting cropped images were resized such that the size of the larva was 70 pixels, to match with the range of 65 – 85 pixels used in generating the training and validation dataset. The scaling parameter for resizing the images was determined by manually calculating the approximate length of the fish (in pixels) for an arbitrary frame and was kept constant for all images. In keeping the scaling parameter constant, we assume that the length of the fish does not change significantly over the dataset. Small variations do not pose an issue due to the tolerance of 20 pixels (65-85 pixels) used in creating the training and validation dataset. A user of our tool may change this range while generating their own training and validation datasets. The resized image was then padded with zeros to obtain a final image resolution of 101x101, which is the required size of the input for the convolutional neural network model described in the previous paragraph. A prediction score for the network’s output is obtained similarly to our 3-D workflow (see **Figure 2**), by rendering physical model images using orthographic projections of the voxel-based model constructed using the pose predictions. (see **Materials and Methods: (b) Template-based pose estimation***: Rendering larval projections*, *Physical model of the larva*) and calculating the correlation coefficient between the rendered physical model image and the input image passed to the convolutional neural network model (see **S2i Figure**).

*3-D pose prediction on 2-camera system: Model training*

We performed pose prediction using our approach on a 2-camera 3-D imaging dataset used in a previous study (4) using a convolutional neural network model. This model was trained on a new training dataset of 500,000 physical model images generated using the ensemble of physical model poses (see **S2d Figure** and **Figure 1a-c**). This network model would accept larval images from the two cameras as the input and generate the respective 12 2-D projection poses coordinates for each of the camera. The imaging system used to record this video dataset (5) consisted two orthogonally aligned cameras, one on the the top of the tank (camera 1, say) and one on the side (camera 2, say). Let ${(x}_{i},y_{i})$ refer to an ordered pair of 2-D pixel coordinate in the frame of camera *i*. The camera axes of telecentric lenses, were aligned such that $x_{1}$ = $x_{2}$ (5). This imaging system is different from ours, which uses fixed focal lenses (see **Materials and Methods: (a) *Instrumentation:*** *Experimental setup and data* collection). and presents a significantly different projection function (see **Materials and Methods: (a) Intrumentation**: *Camera calibration: Mapping from lab to camera coordinates*. As a result, the relation between larval images seen in camera 1 and camera 2 that the network would learn during training, is also significantly different from the implicit relation that exists in our imaging setup. The new training dataset of physical model images had to be created using an appropriate projection function that represented this coupling between the two camera images in the two-camera imaging system (5). We model the projection functions $x_{i}= f_{ix}(x_{lab},y_{lab},z_{lab})$ and $y_{i}= f_{iy}(x_{lab},y_{lab},z_{lab})$ (see **Materials and Methods: (a) *Instrumentation:*** *Experimental setup and data* for references to notations and **S2c Figure**) using a translation and scaling. Specifically, we used the relation $x_{i}=(t_{i}+ s_{i}x_{lab})$ and relation $y_{i}=(t_{i}+ s_{i}x_{lab})$. The scaling parameter $s_{i}$ represents the constant magnification of the telecentric lenses and the translation parameter $t_{i}$ maps the center of the lab reference frame (*x*_lab_, *y*_lab_, *z*_lab_) to the center of the images in either of the cameras. The training dataset was generated using the ensemble of physical model poses that we estimated for our 3-D pose estimation workflow (see **Results: Convolutional neural network model trained on physical model images performs fast and accurate pose estimation on real images***: Generation of the training and validation datasets* and *Ensemble of physical model poses in* **Figure 1c**). Larval length was sampled from a normal distribution centered at 3.8 mm with a standard deviation $\sigma=0.1$. The scaling parameters $s_{i}$ were set such that the length of a 3.8 mm larva as seen in the 2-D pixel coordinates matched the larval length in pixels as seen in the preprocessed images (see the discussion below in **Materials and Methods: (d) Modeling external datasets**: *3-D pose prediction on 2-camera system: Pose prediction*). We generated 500,000 physical model images (see **S2f Figure**) and split them in a 9:1 ratio to obtain a training and validation dataset to train the convolutional neural network model (see **S2g Figure**). The convolutional neural network model used had a similar architecture as that used in all our other implementations: an encoder consisting of four ResNet blocks and a decoder projecting the output pose. The ResNet blocks had 32, 64, 128 and 256 layers, while the decoder consisted of three fully connected layers of dimensions 1x288, 1x144 and 1x48. The output was reshaped into two vectors, each having dimensions 2x12, representing the 2-D projection pose coordinates (10 bacbone coordinates and 2 eye coordinates) for the larval images in the two camera views. Leaky rectinlinear units and batch normalizations were used as described for previously implementations of the convolutional neural network. The network was trained for 100 epochs.

*3-D pose prediction on 2-camera system: Pose prediction*

Raw video frames in (5) were obtained by contacting the authors. Preprocessing of the raw video frames involved background subtraction, median filtered and cropping using a bounding box around the larva (see **S2a,b Figure** and **S7 Figure**). The cropped images was resized to a final resolution of 141x141. Two such preprocessed 141x141 images obtained from camera 1 and camera 2 were passed as the input the convolutional neural network model to obtain a 2 2x12 dimensional arrays (see **S2h Figure**). The set of two 2-D pose projection coordinates, one for each camera view, were mapped to 12 3-D pose coordinates (10 3-D backbone coordinates and 2 3-D centroid coordinates of the eyes) of the larva (see **Convolutional neural network model trained on physical model images performs fast and accurate pose estimation on real images**: ***The 3-D physical model of a larva***). This 2-D to 3-D mapping is done by inverting the pose projection functions discussed previously in **Materials and Methods: (d) Modeling external datasets:** *3-D pose prediction on 2-camera system: Model training*. Note that $x_{lab}$ can be obtained either from $x_{1}$ or $x_{2}$. Since the fits to camera 1 were seen to be consistently better than those to camera 2, we used $x_{1}$ to infer $x_{lab}$. Alternatively, one can also infer $x_{lab}$ independently using $x_{1}$ and $x_{2}$, and calculate their mean. Pose prediction score was calculated by rendering the physical model corresponding to the inferred 3-D pose coordinates from the neural network and compting the correlation coefficient between the physical model image to the cropped preprocessed image used as the network model’s input (see **S2i Figure**).

**References**

1. He K, Zhang X, Ren S, Sun J. Deep Residual Learning for Image Recognition. In: 2016 IEEE Conference on Computer Vision and Pattern Recognition (CVPR) [Internet]. Las Vegas, NV, USA: IEEE; 2016 [cited 2022 Oct 7]. p. 770–8. Available from: http://ieeexplore.ieee.org/document/7780459/

2. Xu B, Wang N, Chen T, Li M. Empirical Evaluation of Rectified Activations in Convolutional Network [Internet]. arXiv; 2015 [cited 2022 Nov 13]. Available from: http://arxiv.org/abs/1505.00853

3. Ioffe S, Szegedy C. Batch Normalization: Accelerating Deep Network Training by Reducing Internal Covariate Shift. :9.

4. Bolton AD, Haesemeyer M, Jordi J, Schaechtle U, Saad FA, Mansinghka VK, et al. Elements of a stochastic 3D prediction engine in larval zebrafish prey capture. Berman GJ, Calabrese RL, Washbourne P, Combes SA, editors. eLife. 2019 Nov 26;8:e51975.

5. Elements of a stochastic 3D prediction engine in larval zebrafish prey capture | eLife [Internet]. [cited 2023 Jun 19]. Available from: https://elifesciences.org/articles/51975
