# Supplementary material for: Rapid automated 3-D pose estimation of larval zebrafish using a physical model-trained neural network
Source: PLoS Comput Biol. 2023 Oct 23;19(10):e1011566. doi: 10.1371/journal.pcbi.1011566 (PMC10621986; doi:10.1371/journal.pcbi.1011566)
Supplement: S1 Table — The extrinsic matrices M shown below encode the orientation and location of the cameras, where the translation vector T is estimated in mm. The intrinsic matrices K encode the intrinsic parameters of the camera in pixels (1 pixel = 7.4 μm). The two matrices were used to analyze fish swimming experiments and had to be calibrated periodically to account for drift in the cameras. (DOCX) [file pcbi.1011566.s002.docx]

**S1 Table:** **Camera calibration parameters:** Intrinsic and extrinsic matrices illustrating the camera parameters used for analysis. The extrinsic matrices **M** shown below encode the orientation and location of the cameras, where the translation vector T is estimated in mm. The intrinsic matrices **K** encode the intrinsic parameters of the camera in pixels (1 pixel = 7.4 μm). The two matrices were used to analyze fish swimming experiments and had to be calibrated periodically to account for drift in the cameras.

| **Camera** | **Intrinsic Matrix**  **K =** $\left[ \begin{matrix} f_{x} & 0 & 0 \\ \gamma& f_{y} & 0 \\ \mu_{0} & \nu_{0} & 1 \end{matrix} \right]$ | **Extrinsic Matrix**  **M =** $\left[ \begin{matrix} R_{3x3} \\ T_{1x3} \end{matrix} \right]$ |
| --- | --- | --- |
| 1 | $\left[ \begin{matrix} 1143.2 & 0 & 0 \\ 0 & 1139.4 & 0 \\ 327.2 & 233.2 & 1 \end{matrix} \right]$ | $\left[ \begin{matrix} 0.82 & -0.57 & -0.02 \\ 0.40 & 0.61 & -0.68 \\ 0.40 & 0.55 & 0.73 \\ -16.98 & -0.07 & 81.37 \end{matrix} \right]$ |
| 2 | $\left[ \begin{matrix} 1133.4 & 0 & 0 \\ 0 & 1138.5 & 0 \\ 345.9 & 244.1 & 1 \end{matrix} \right]$ | $\left[ \begin{matrix} 0.56 & 0.02 & 0.83 \\ 0.62 & -0.67 & -0.4 \\ 0.55 & 0.74 & -0.40 \\ -12.14 & 1.21 & 80.63 \end{matrix} \right]$ |
| 3 | $\left[ \begin{matrix} 1127.7 & 0 & 0 \\ 0 & 1129.7 & 0 \\ 329.2 & 248.6 & 1 \end{matrix} \right]$ | $\left[ \begin{matrix} 0.82 & 0.03 & -0.57 \\ 0.40 & -0.67 & 0.62 \\ 0.40 & -0.74 & 0.54 \\ -17.44 & -11.04 & 87.53 \end{matrix} \right]$ |
