# Supplementary material for: Rapid automated 3-D pose estimation of larval zebrafish using a physical model-trained neural network
Source: PLoS Comput Biol. 2023 Oct 23;19(10):e1011566. doi: 10.1371/journal.pcbi.1011566 (PMC10621986; doi:10.1371/journal.pcbi.1011566)
Supplement: S2 Table — (DOCX) [file pcbi.1011566.s003.docx]

**S2 Table: Comparison of convolutional neural network model pose predictions on different datasets:** Convolutional neural network models trained on different datasets and average pose prediction scores on real data from the three experiments combined.

| **Dataset** | 100% | 25% | Free swimming | Acoustic Startle | Dark Flash | Template-based | Wider kernel^a^ | Two-camera^b^ |
| --- | --- | --- | --- | --- | --- | --- | --- | --- |
| **Mean prediction score** | 0.91 | 0.91 | 0.90 | 0.91 | 0.91 | 0.90 | 0.92 | 0.93 |

^a^Network model trained on an ensemble of physical model poses generated using a kernel bandwidth 2x wider than used elsewhere.

^b^Network model trained on a fictitious two-camera imaging system using our projection function.
