# Supplementary material for: Rapid automated 3-D pose estimation of larval zebrafish using a physical model-trained neural network
Source: PLoS Comput Biol. 2023 Oct 23;19(10):e1011566. doi: 10.1371/journal.pcbi.1011566 (PMC10621986; doi:10.1371/journal.pcbi.1011566)
Supplement: S3 Table — The upper (lower) left triangle of each sub-table compares the upper (lower) quartiles of each parameter across the three experiments: free swimming, acoustic startle, and dark flash. (DOCX) [file pcbi.1011566.s004.docx]

**S3 Table. p-values from comparing the ranges of different kinematic parameters between different experiments.** The upper (lower) left triangle of each sub-table compares the upper (lower) quartiles of each parameter across the three experiments: free swimming, acoustic startle, and dark flash.

| Max(Δ*z*_0_)   \| **Upper**  **Lower** \| Free  Swimming \| Acoustic  Startle \| Dark  Flash \| \| --- \| --- \| --- \| --- \| \| Free  Swimming \|  \| <0.001 \| <0.001 \| \| Acoustic  Startle \| <0.001 \|  \| <0.001 \| \| Dark  Flash \| <0.001 \| <0.001 \|  \| | Max(<Δ𝜑*_i_*>)   \| **Upper**  **Lower** \| Free  Swimming \| Acoustic  Startle \| Dark  Flash \| \| --- \| --- \| --- \| --- \| \| Free  Swimming \|  \| 0.002 \| <0.001 \| \| Acoustic  Startle \| <0.001 \|  \| 0.44 \| \| Dark  Flash \| <0.001 \| 0.002 \|  \| |
| --- | --- | --- | --- | --- | --- | --- | --- | --- | --- | --- | --- | --- | --- | --- | --- | --- | --- | --- | --- | --- | --- | --- | --- | --- | --- | --- | --- | --- | --- | --- | --- | --- | --- |
| Max(Δ𝜃_0_)   \| **Upper**  **Lower** \| Free  Swimming \| Acoustic  Startle \| Dark  Flash \| \| --- \| --- \| --- \| --- \| \| Free  Swimming \|  \| <0.001 \| <0.001 \| \| Acoustic  Startle \| <0.001 \|  \| 0.40 \| \| Dark  Flash \| <0.001 \| 0.30 \|  \| | Max**(<**Δ𝜃*_i_*>)   \| **Upper**  **Lower** \| Free  Swimming \| Acoustic  Startle \| Dark  Flash \| \| --- \| --- \| --- \| --- \| \| Free  Swimming \|  \| <0.001 \| <0.001 \| \| Acoustic  Startle \| <0.001 \|  \| 0.32 \| \| Dark  Flash \| <0.001 \| 0.18 \|  \| |
| Max**(**Δ𝜑_0_)   \| **Upper**  **Lower** \| Free  Swimming \| Acoustic  Startle \| Dark  Flash \| \| --- \| --- \| --- \| --- \| \| Free  Swimming \|  \| <0.001 \| 0.56 \| \| Acoustic  Startle \| <0.001 \|  \| <0.001 \| \| Dark  Flash \| <0.001 \| <0.001 \|  \| | ℽ_0_   \| **Upper**  **Lower** \| Free  Swimming \| Acoustic  Startle \| Dark  Flash \| \| --- \| --- \| --- \| --- \| \| Free  Swimming \|  \| <0.001 \| 1.0 \| \| Acoustic  Startle \| <0.001 \|  \| 0.32 \| \| Dark  Flash \| <0.001 \| <0.001 \|  \| |
| Max(Δℽ_0_)   \| **Upper**  **Lower** \| Free  Swimming \| Acoustic  Startle \| Dark  Flash \| \| --- \| --- \| --- \| --- \| \| Free  Swimming \|  \| <0.001 \| 0.55 \| \| Acoustic  Startle \| <0.001 \|  \| 0.025 \| \| Dark  Flash \| <0.001 \| 0.079 \|  \| | 𝜑_0_   \| **Upper**  **Lower** \| Free  Swimming \| Acoustic  Startle \| Dark  Flash \| \| --- \| --- \| --- \| --- \| \| Free  Swimming \|  \| <0.001 \| 1.0 \| \| Acoustic  Startle \| <0.001 \|  \| <0.001 \| \| Dark  Flash \| 1.0 \| <0.001 \|  \| |
