# Supplementary figures and images for: Rapid automated 3-D pose estimation of larval zebrafish using a physical model-trained neural network
Source: PLoS Comput Biol. 2023 Oct 23;19(10):e1011566. doi: 10.1371/journal.pcbi.1011566 (PMC10621986; doi:10.1371/journal.pcbi.1011566)

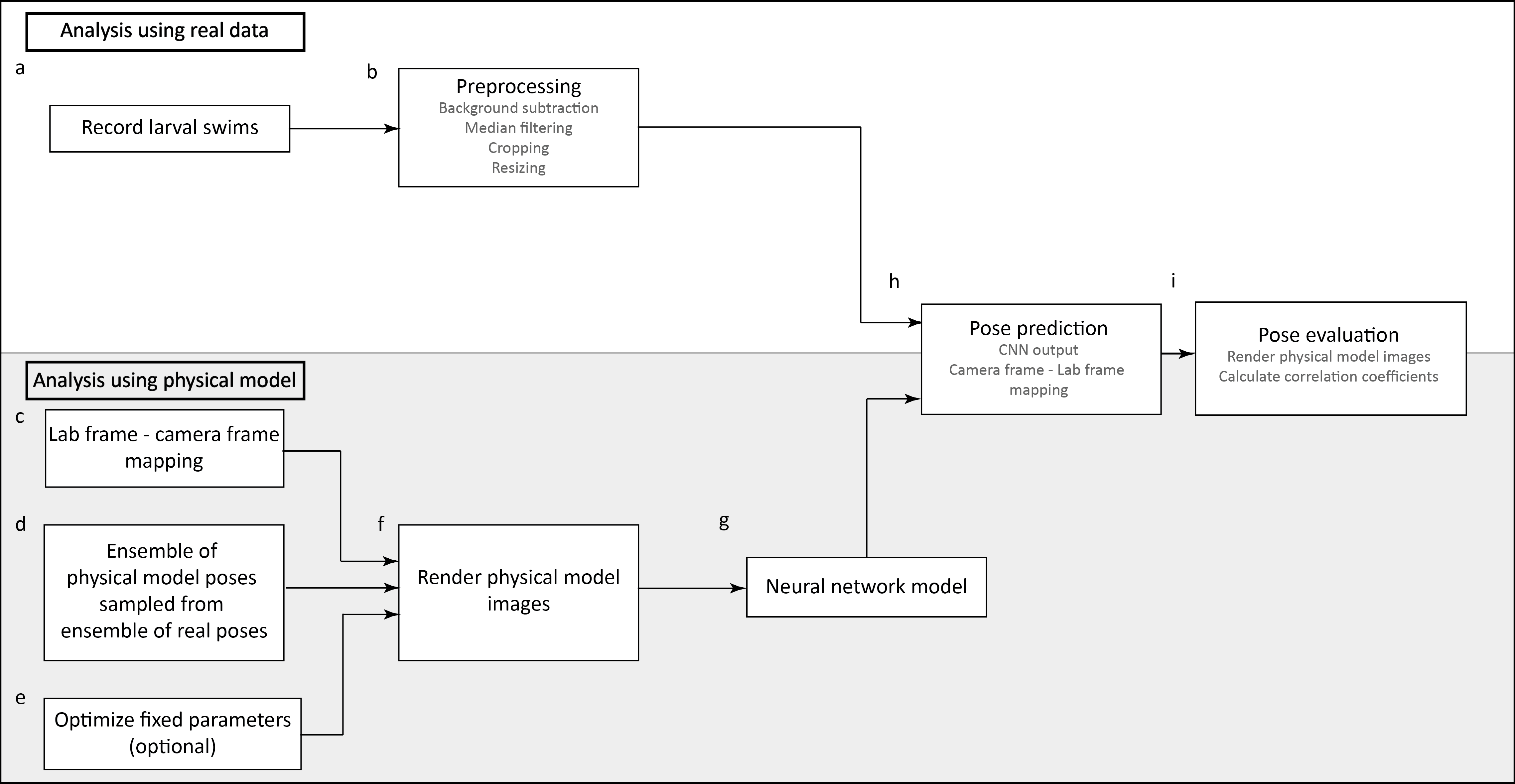

Supplement: S2 Fig — (a) Video recordings of larval behavior are obtained using a 2-D or 3-D imaging system. (b) Larval images are preprocessed: Background subtraction: The raw image is subtracted from the background image. Median filtering: A median filter of kernel size [5,5] is convolved over the image to reduce noise. Cropping: A bounding box is cropped around the larva. Resizing: The cropped image is resized so that the length of the larva is within the range of lengths used to generate the physical model images in the training dataset. (c) A projection function mapping the 3-D lab frame coordinates to 2-D pixel coordinates is empirically determined. (d) An ensemble of physical models is sampled using the ensemble of real larval poses. We have generated a representative ensemble of real poses for 2-D and 3-D larval motion. The appropriate ensemble must be selected based on the imaging setup (2-D or 3-D) used in (a). (e) The fixed parameters of the physical model are estimated. We have shown that this step is not a strict requirement as training datasets generated with a certain set of fixed parameters generalize for pose prediction on datasets in different imaging conditions. (f) A training dataset of physical model images and their underlying pose annotations is generated. (g) A convolutional neural network model is trained to learn the relationship between physical model images and the 2-D projection pose coordinates. (h) Pose prediction is performed by passing the preprocessed images (b) as input to the convolutional neural network model. The larval coordinates in the lab reference frame are evaluated using the 2-D projection pose coordinates using a mapping from camera frame to lab frame. (i) Pose evaluation is performed by comparing the physical model images resulting from the neural network’s predicted 3-D pose and the preprocessed input in (b) passed as input to the trained network model. The comparison is quantitatively computed as Pearson’s correlation coefficient. (PNG) [file pcbi.1011566.s006.png]

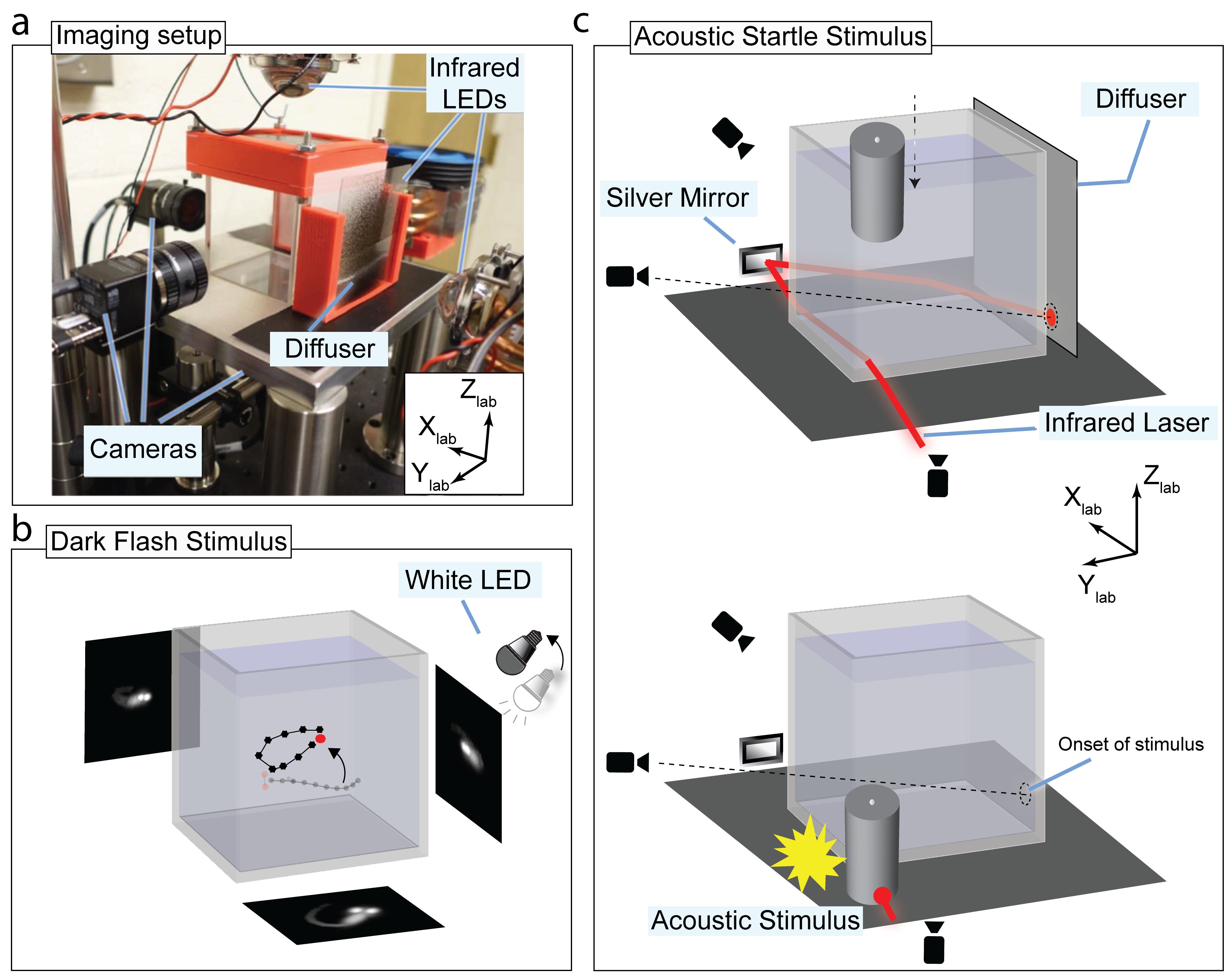

Supplement: S3 Fig — (a) Experimental setup for 3-D experiments. Zebrafish larvae swimming freely in a 7 x 7 x 7 cm glass tank are imaged using three cameras at 500 fps under infrared illumination. (b) Schematic of the setup used to provide acoustic stimulus. Larvae are habituated to white light using a white LED for at least 4 minutes. A dark flash stimulus is provided when the three cameras simultaneously detect a unique larva in their field of view. The instantaneous deactivation of the white LED marks the onset of the dark flash stimulus. (c) Schematic of the setup used to provide acoustic stimulus. The stimulus is generated by dropping a cylindrical weight on the platform supporting the tank from a suitable height. The acoustic signal propagating through the cube startles the larva. The time of the stimulus is recorded using an infrared laser projected in the field of view of one of the cameras. The weight is pulled back up using an Arduino-controlled servo motor and held in place using an electromagnet until the next round of stimulus. (PNG) [file pcbi.1011566.s007.png]

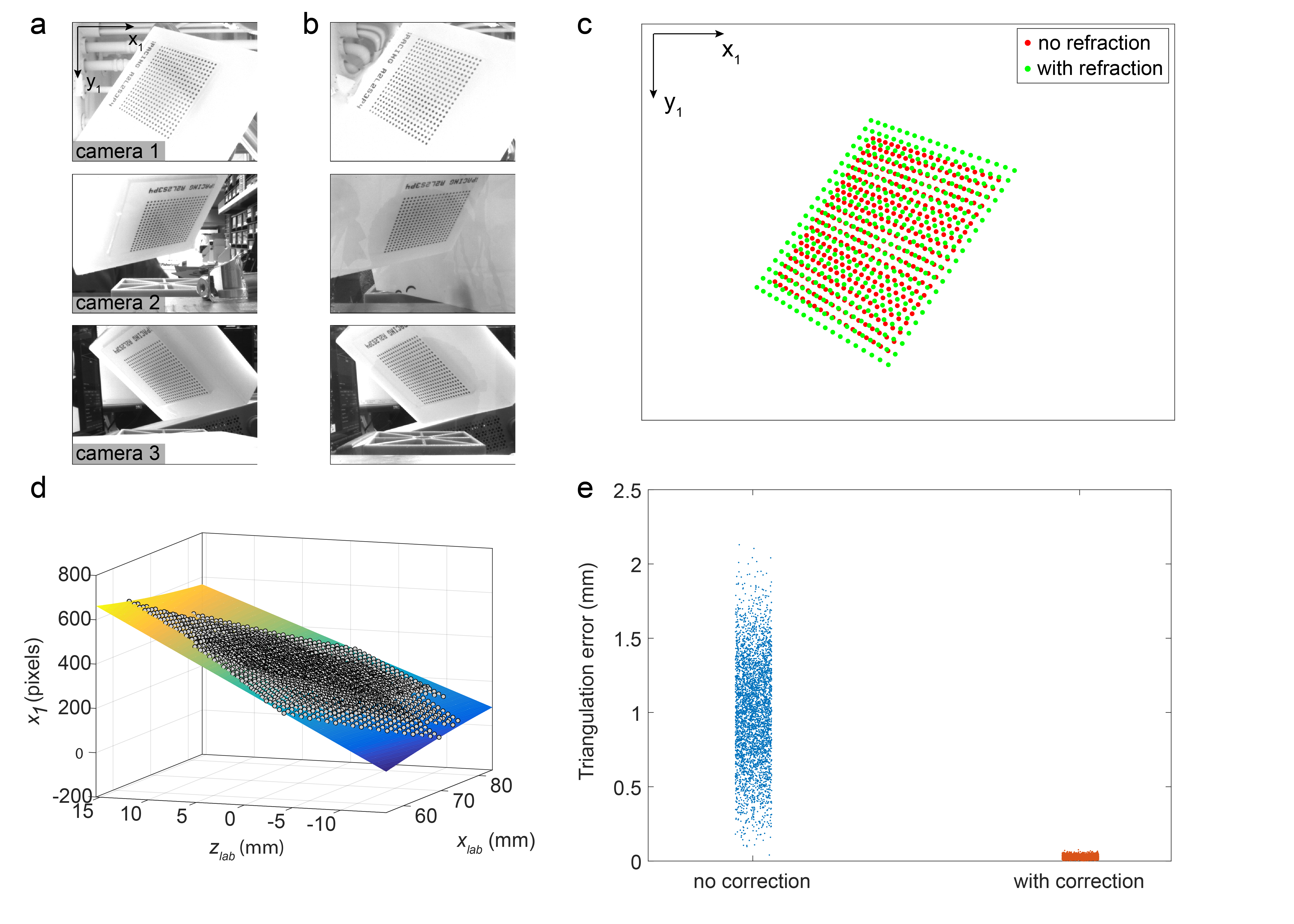

Supplement: S4 Fig — (a) Images of the calibration dot pattern in air. (b) Images of the same calibration pattern inside the glass cube filled with water. The position of the pattern is identical in both sets of images. (c) A sample image showing the coordinates of the dots on the images taken by one of the cameras. Green dots correspond to an image in (a) (camera 1), in the absence of refraction. Red dots correspond to an image in (b) (camera 1), in the presence of refraction from the glass cube and water. (d) The projection function (f1x(ylab, zlab)) for the coordinates x1 in camera 1 of the dot pattern, and fit to a cubic function. The surface plot color represents x1 for visual convenience. (e) Triangulation error of the lab coordinates of the dots on the calibration pattern from the images taken in water. The mean error is 1.07 mm without refraction correction and 0.03 mm with correction. (PNG) [file pcbi.1011566.s008.png]

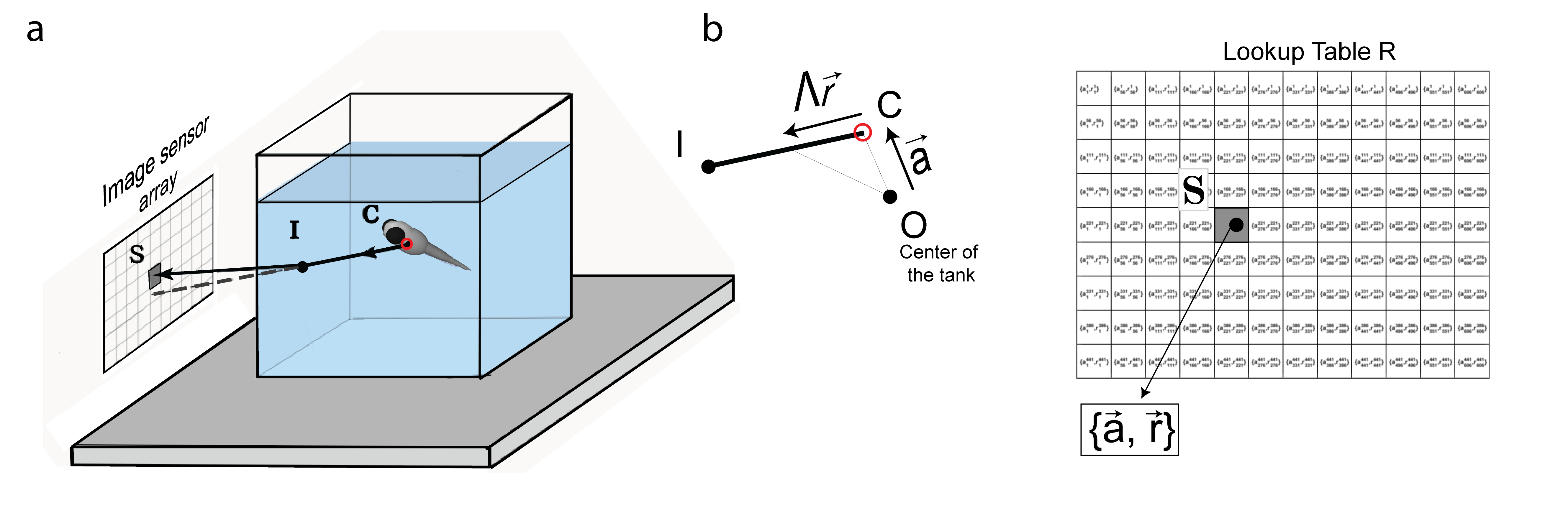

Supplement: S5 Fig — (a) Ray CI emanates from the centroid of the larva, C, and passes through an arbitrary point I in the tank. By invoking ray optics, for every pixel S of the camera sensor array, there is a unique ray CI emanating from a point C inside the tank that is incident on S after refraction at the water-tank-air interface. (b) Lookup Table R stores the two vectors a and r that uniquely define ray CI for every pixel of the camera sensor, for all three cameras. (PNG) [file pcbi.1011566.s009.png]

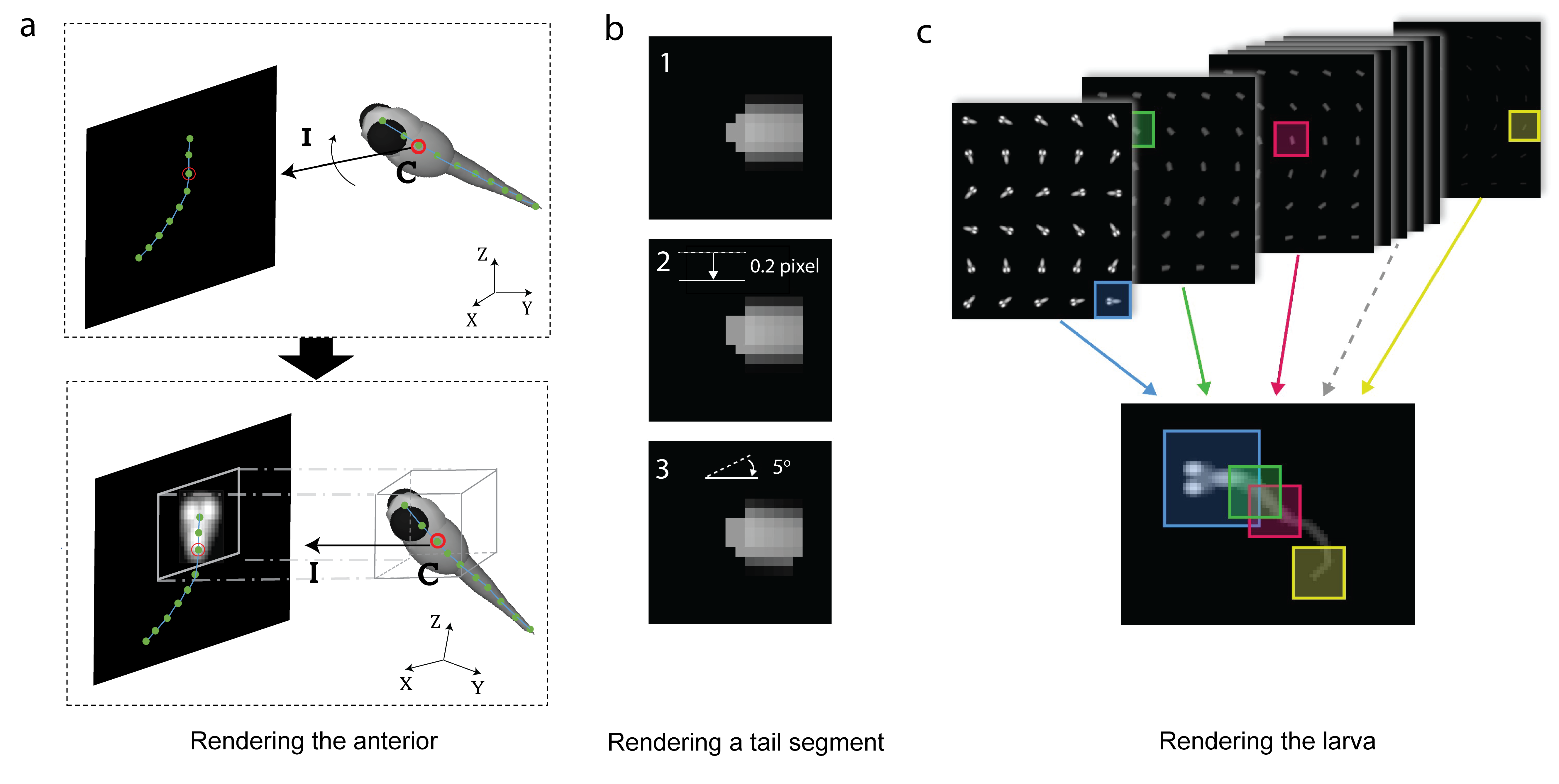

Supplement: S6 Fig — (a) Rendering the larval anterior. Lookup Table P entries of the anterior are generated using orthographic projections of a voxel-based model along the principal axis of each camera. Given the parameter vector p, the indices of the appropriate lookup table entry are determined to render the digital projection. The 2-D projection of the larval backbone (green chain) is computed using the nonlinear projection parameters (top panel). Ray CI emanates from the 3-D larva’s centroid and is incident on its 2-D projection. The appropriate lookup table entry to be used is determined by rotating CI such that it is parallel to the principal axis of the corresponding camera (bottom panel). (b) Rendering a tail segment. Image 1 shows a digitally rendered component of a tail segment. Images 2 and 3 show entries in the lookup table adjacent to the entry for image 1. The tail segment in image 2 is 0.2 pixels lower than that in image 1. The segment in image 3 is that in image 1 rotated by 5° clockwise. (c) Rendering the larva. Scheme for the construction of a grayscale fish model from the lookup table. Only a small fraction of the tables is shown. (PNG) [file pcbi.1011566.s010.png]

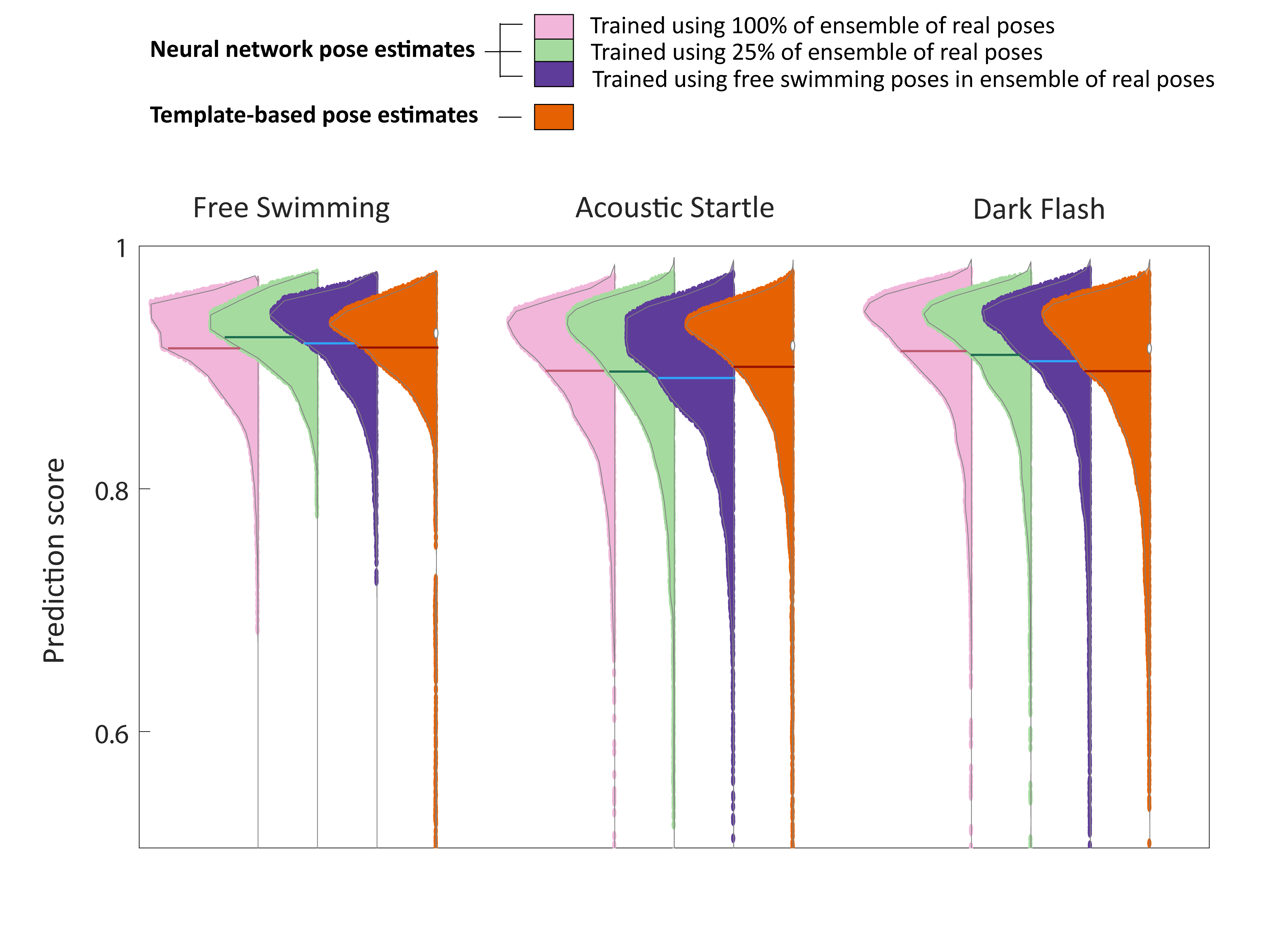

Supplement: S8 Fig — The distributions of predictions scores are visualized using kernel density estimate over pose prediction scores obtained using different approaches across free swimming, acoustic startle and dark flash experiments. The distributions compare pose prediction accuracy of network models trained using different subsets of the ensemble of real poses and that of the physical model fits. The mean of each distribution is shown using overlaid dashed lines. Using only 25% (N = 8929) of poses from the ensemble of real poses to generate the training dataset does not deteriorate network predictions, compared to the performance of a network trained using 100% (N = 35714) of poses from the ensemble of real poses (compare pink and green distributions and their means–overlaid dashed lines). When the training dataset is generated using only free swimming poses from the ensemble of real poses (N = 9536), the network performance for acoustic startle and dark flash experiments declines marginally (purple distributions and overlaid dashed lines). The template-based pose estimates are marginally better than neural network predictions in the acoustic startle experiments and consistently worse in dark flash experiments. On average, the network trained using 100% of the data (mean prediction score = 0.91) is comparable to the template-based predictions (mean predictions score = 0.90). (PNG) [file pcbi.1011566.s012.png]

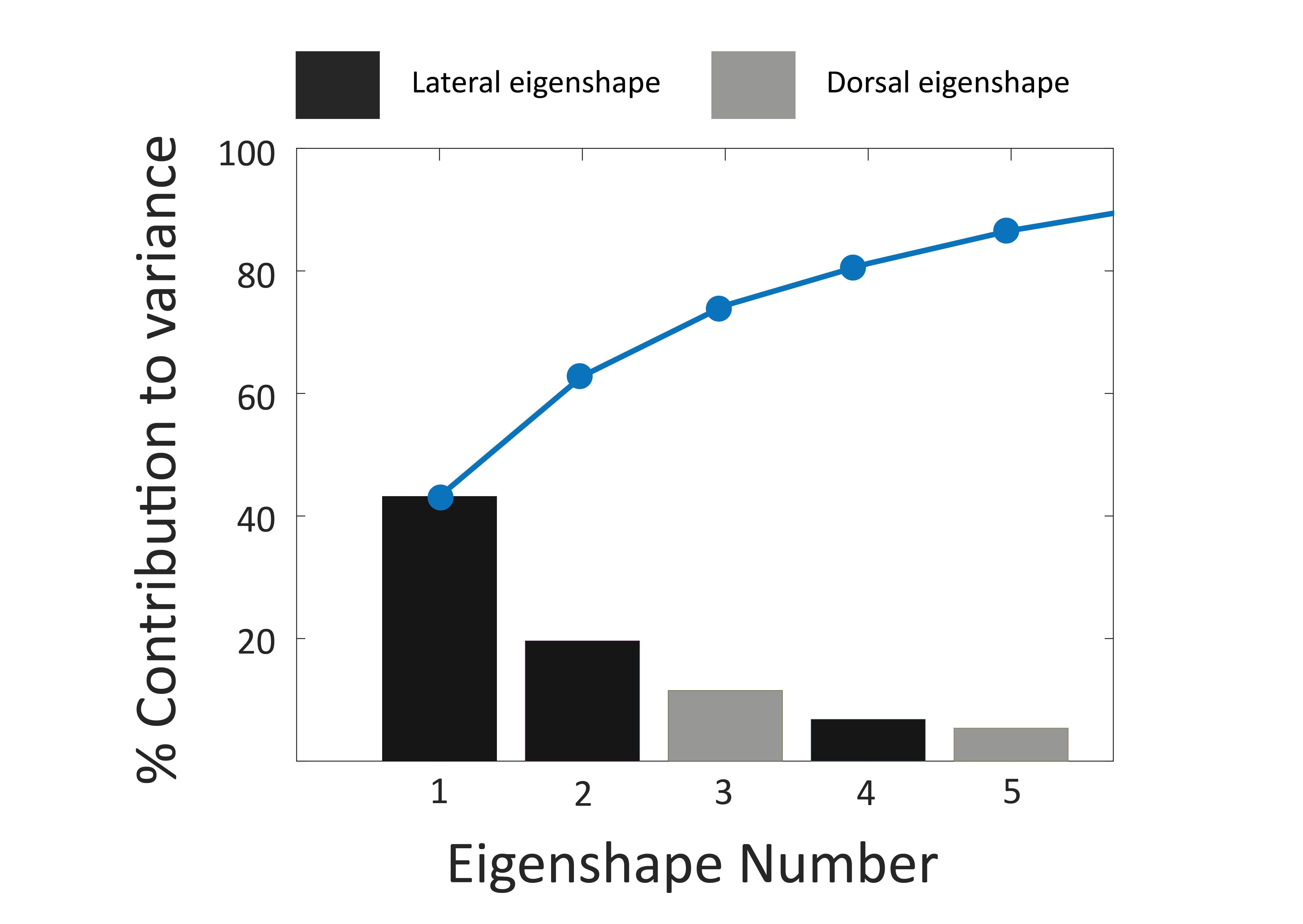

Supplement: S10 Fig — Singular Value Decomposition of ϴ(t) from all swim bouts identifies orthogonal bending modes or “eigenshapes” of the larvae (see Ref. 23). Eigenshapes are arranged in decreasing order of their contribution to the total variance in ϴ(t). Eigenshapes 1, 2, and 4 (Lateral Eigenshapes—black) mainly contribute to lateral bending motion (Δφi ~ 0), while eigenshapes 3 and 5 (Dorsal Eigenshapes—gray) mainly contribute to dorso-ventral bending motion (Δθi ~ 0). The blue curve indicates the cumulative contribution of the first several eigenshapes to the total variance in ϴ(t). (PNG) [file pcbi.1011566.s014.png]

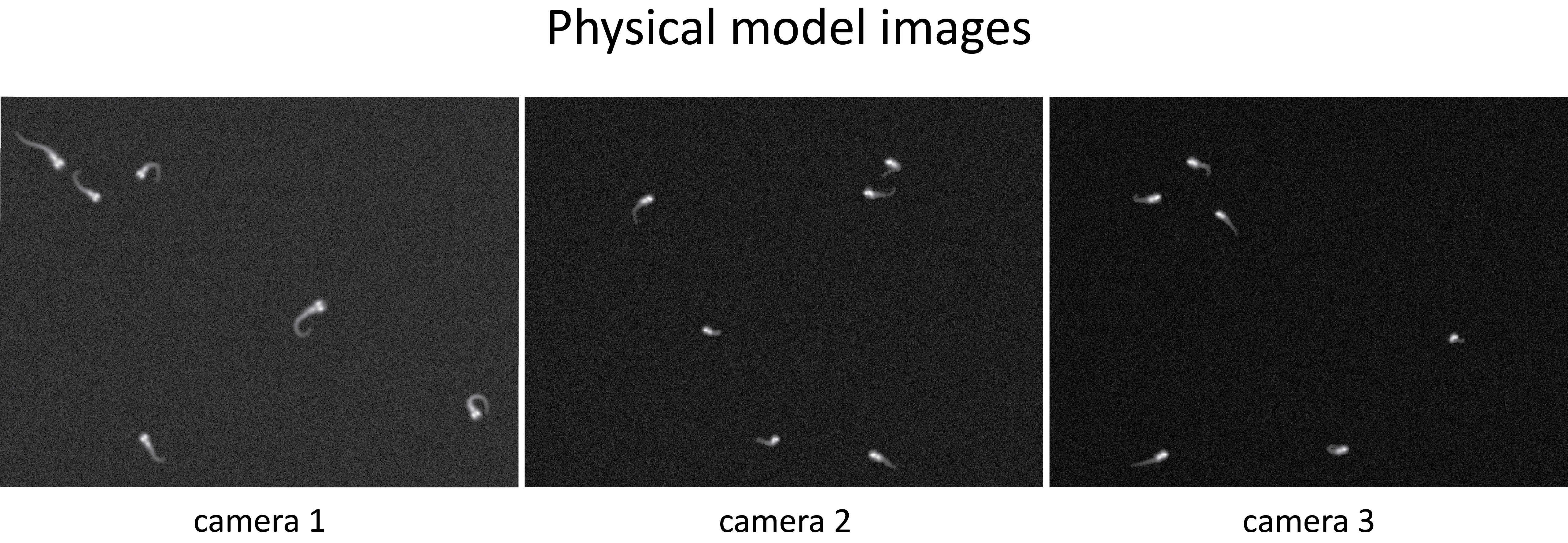

Supplement: S11 Fig — Physical model projections of multiple larvae are rendered as an extension of our approach. A large dataset of such images can be potentially used to develop pose prediction workflows to study social behavior of larval zebrafish. The network architecture and loss function used for such a workflow when fish number varies from frame to frame may be adapted from successful models developed for multi-animal pose estimation task (59–61). (PNG) [file pcbi.1011566.s015.png]
